# Supplementary material for: Global Cutaneous Mucormycosis: A Systematic Review
Source: J Fungi (Basel). 2022 Feb 16;8(2):194. doi: 10.3390/jof8020194 (PMC8878367; doi:10.3390/jof8020194)
Supplement: Supplementary file 1 [file jof-08-00194-s001.zip › jof-1592643-supplementary.pdf]

File S1. Articles included in the analysis for the systematic review

1. Adam, R.D; Hunter, G.; Di Tomasso, J.; Comerci, G.Jr. Mucormycosis: emerging prominence of cutaneous infections. *Clin. Infect. Dis*. **1994**, *19*, 67-76.
2. Ahmadinejad, Z.; Khazraiyan, H.; Ghanbari, F.; Ahmadi, B.; Shoar, M.G. Cutaneous mucormycosis in a diabetic patient following traditional dressing. *Case Rep. Dermatol. Med.* **2013**, 2013:894927.
3. Ajello, L.; Dean, D.F.; Irwin, R.S. The zygomycete *Saksenaea vasiformis* as a pathogen of humans with a critical review of the etiology of zygomycosis. *Mycologia* **1976**, *68*, 52-62.
4. Akritidis, N.; Papaioannides, D.; Kitsiou, E.; Korantzopoulos, P. Necrotizing cutaneous mucormycosis. *Hospital Medicine* **2002**, *63*, 308-9.
5. Al-Hedaithy, M. Cutaneous zygomycosis due to *Saksenaea vasiformis*: case report and literature review. *An. Saudi Med*. **1998,** *18*, 428-31.
6. Al-Ajam, M.R.; Bizri, A.R.; Mokhbat, J.; Weedon, J.; Lutwick, L. Mucormycosis in the Eastern Mediterranean: a seasonal disease. *Epidemiol. Infect*. **2006**, *134*, 341-6.
7. Al-Tarrah, K.; Abdelaty, M.; Behbahani, A.; Mokaddas, E.; Soliman, H.; Albader, A. Cutaneous mucormycosis postcosmetic surgery: a case report and review of the literature. *Medicine* **2016**, *95*, 27.
8. Al-Zaydani, I.A.; Al-Hakami, A.M.; Joseph, M.R.; Kassem, W.M.; Almaghrabi, M.K.; Nageeb, A.; Hamid, M.E. Aggressive cutaneous zygomycosis caused by *Apophysomyces variabilis* in an immunocompetent child. *Med. Mycol. Case Rep*. **2015**, *10*, 11-3.
9. Albízuri-Prado, M.F.; Sánchez-Orta, A.; Rodríguez-Bandera, A.; Feito-Rodríguez, M. Primary cutaneous mucormycosis Due to *Rhizopus arrhizus* in an 8-year-old girl. *Actas Dermosifiliogr*. **2017**, *109*, 562-4.

10. Almaslamani, M.; Taj-Aldeen, S.J.; Garcia-Hermoso, D.; Dannaoui, E.; Alsoub, H.; Alkhal, A. An increasing trend of cutaneous zygomycosis caused by *Mycocladus corymbifer* (formerly *Absidia corymbifera*): report of two cases and review of primary cutaneous *Mycocladus* infections. *Med. Mycol*. **2009**, *47*, 532-8.
11. Al-Qattan, M.M.; Al Mazrou, A.M. Mucormycosis of the upper limb. *J. Hand Surg*. **1996**, *21*, 261-2.
12. Alseady, A.; Baharoon, S. Acute cutaneous zygomycosis of the scalp: A case report and literature review. *J. Infect. Public Health* **2015**, *8*, 377-81.
13. Alsuwaida, K. Primary cutaneous mucormycosis complicating the use of adhesive tape to secure the endotracheal tube. *Can. J. Anesth*. **2002**, *49*, 880-2.
14. Amatya, R.; Khanal, B.; Rijal, A. *Syncephalastrum* species producing mycetoma-like lesions. *Indian J. Dermatol. Venereol. Leprol*. **2010**, *76*, 284-6.
15. Ameen, M.; Arenas, R.; Martinez-Luna, E.; Reyes, M.; Zacarias, R. The emergence of mucormycosis as an important opportunistic fungal infection: five cases presenting to a tertiary referral center for mycology. *Int. J. Dermatol*. **2007**, *46*, 380-4.
16. Amin, S.B.; Ryan, R.M.; Metlay, L.A.; Watson, W.J. *Absidia corymbifera* infections in neonates. *Clin. Infect. Dis*. **1998**, *26*, 990-2.
17. Andresen, D.; Donaldson, A.; Choo, L.; Knox, A.; Klaassen, M.; Ursic, C.; Vonthethoff, L.; Krilis, S.; Konecny, P. Multifocal cutaneous mucormycosis complicating polymicrobial wound infections in a tsunami survivor from Sri Lanka. *Lancet* **2005**, *365*, 876-8.
18. Antonov, N.K.; Tang, R.; Grossman, M.E. Utility of touch preparation for rapid diagnosis of cutaneous mucormycosis. *JAAD Case Rep*. **2015**, *1*, 175.

19. Arisoy, A.E.; Arisoy, E.S.; Correa-Calderon, A.; Kaplan, S.L. *Rhizopus* necrotizing cellulitis in a preterm infant: a case report and review of the literature. *Pediatr. Infect. Dis. J.* **1993**, *12*, 1029-31.
20. Arnáiz-García, M.E.; Alonso-Peña, D.; del Carmen González-Vela, M.; García-Palomo, J.D.; Sanz-Giménez-Rico, J.R.; Arnáiz-García, A.M. Cutaneous mucormycosis: report of five cases and review of the literature. *J. Plast. Reconstr. Aesthet. Surg*. **2009**, *62*, e434-41.
21. Ashkenazi-Hoffnung, L.; Bilavsky, E.; Avitzur, Y.; Amir, J. Successful treatment of cutaneous zygomycosis with intravenous amphotericin B followed by oral posaconazole in a multivisceral transplant recipient. *Transplantation* **2010**, *90*, 1133-5.
22. Austin, C.L.; Finley, P.J.; Mikkelson, D.R.; Tibbs, B. Mucormycosis: a rare fungal infection in tornado victims. *J. Burn Care Res*. **2014**, *35,* e164-71.
23. Ayala-Gaytán, J.J.; Petersen-Morfín, S.; Guajardo-Lara, C.E.; Barbosa-Quintana, A.; Morfín-Otero, R.; Rodriguez-Noriega, E. Cutaneous zygomycosis in immunocompetent patients in Mexico. *Mycoses* **2009**, *53*, 538-40.
24. Aziz, S.; Merrell, R.C.; Edwards, M.F. Mucormycosis in patients with multiple-organ failure. *Arch. Surg*. **1984**, *119*, 1189-91.
25. Bandettini, R.; Tuo, P.; Tamisani, A.M.; Di Marco, E.; Mantero, E. Severe cutaneous zygomycosis: evaluation of treatment with high doses of liposomal amphotericin B in a one-year old child. *J. Chemother*. **2007**, *19*, 347-9.
26. Baradkar, V.P.; Kumar, S. Cutaneous zygomycosis due to *Saksenaea vasiformis* in an immunocompetent host. *Indian J. Dermatol*. **2009**, *54(4),* 382-4.

Articles included in the analysis for the systematic review

27. Baraia, J.; Munoz, P.; de Quirós, J.B.; Bouza, E. Cutaneous mucormycosis in a heart transplant patient associated with a peripheral catheter. *Eur. J. Clin. Microbiol. Infect. Dis.* **1995**, *14*, 813-5.
28. Bateman, C.P.; Umland, E.T.; Becker, L.E. Cutaneous zygomycosis in a patient with lymphoma. *J. Am. Acad. Dermatol*. **1983**, *8*, 890-4.
29. Bearer, E.A.; Nelson, P.R; Chowers, M.Y.; Davis, C.E. Cutaneous zygomycosis caused by *Saksenaea vasiformis* in a diabetic patient. *J. Clin. Microbiol*. **1994**, *32,* 1823-4.
30. Beatty, N.; Al Mohajer, M. Primary cutaneous mucormycosis developing after incision and drainage of a subcutaneous abscess in an immunocompetent host. *BMJ Case Rep*. **2016**, bcr2015213700.
31. Becker, B.C.; Schuster, F.R.; Ganster, B.; Seidl, H.P.; Schmid, I. Cutaneous mucormycosis in an immunocompromised patient. *Lancet Infect. Dis*. **2006**, *6*, 536.
32. Belfiori, R.; Terenzi, A.; Marchesini, L.; Repetto, A. *Absidia corymbifera* in an immune competent accident victim with multiple abdominal injuries: case report. *BMC Infect. Dis*. **2007**, *7*, 1-5.
33. Bellazreg, F.; Hattab, Z.; Meksi, S.; Mansouri, S.; Hachfi, W.; Kaabia, N.; Said, M.B.; Letaief, A. Outcome of mucormycosis after treatment: report of five cases. *New Microbes New Infect*. **2015,** *6*, 49-52.
34. Belliere, J.; Rolland, M.; Tournier, E.; Cassaing, S.; Iriart, X.; Paul, C.; Kamar, N. Early necrotic skin lesions after an ABO-incompatible kidney transplantation: The threat of *Cunninghamella* spp. *Transpl. Infect. Dis*. **2019**, *21*, e13173.
35. Benjamin, T.; Wattier, R.; Dominic, W. Allograft of primary cutaneous mucormycosis in a preterm neonate: a case report. *Wounds* **2019**, 31, E46-8.

36. Bentur, Y.; Shupak, A.; Ramon, Y.; Abramovich, A.; Wolfin, G.; Stein, H.; Krivoi, N. Hyperbaric oxygen therapy for cutaneous/soft-tissue zygomycosis complicating diabetes mellitus. *Plast. Reconstr. Surg*. **1998**, *102*, 822-4.
37. Berot, V.; Bernigaud, C.; Ferchiou, A.; Ingen-Housz-Oro, S.; Hüe, S.; Ajzenberg, C.; Thomas, S.; et al. Extensive cutaneous and muscular mucormycosis complicating insulin pump treatment. *J. Eur. Acad. Dermatol. Venereol*. **2020**, *34*, e486-9.
38. Bezzant, M.; Lago, K.; Calvano., T. Case report of penile *Rhizopus arrhizus* infection in an unexpected patient. *Med. Mycol. Case Rep.* **2019**, *26*, 16-8.
39. Bibashi, E.; de Hoog, G.S.; Pavlidis, T.E.; Symeonidis, N.; Sakantamis, A.; Walther, G. Wound infection caused by *Lichtheimia ramosa* due to a car accident. *Med. Mycol. Case Rep*. **2013**, *2,* 7-10.
40. Blair, J.E.; Fredrikson, L.J.; Pockaj, B.A.; Lucaire, C.S. Locally invasive cutaneous *Apophysomyces elegans* infection acquired from snapdragon patch test. *Mayo Clin. Proc.* **2002**, 77, 717-20.
41. Blanchet, D.; Dannaoui, E.; Fior, A.; Huber, F.; Couppié, P.; Salhab, N.; *et al*. *Saksenaea vasiformis* infection, French Guiana. *Emerg. Infect. Dis.* **2008**, *14*, 342.
42. Blazquez, D.; Ruiz-Contreras, J.; Fernández-Cooke, E.; González-Granado, I.; Delgado, M.D.; Menendez, M.T.; *et al. Lichtheimia corymbifera* subcutaneous infection successfully treated with amphotericin B, early debridement, and vacuum-assisted closure. *J. Pediatr. Surg*. **2010**, *45*, e13-5.
43. Bonifaz, A.; Stchigel, A.M.; Guarro, J.; Guevara, E.; Pintos, L.; Sanchis, M.; Cano-Lira, J.F. Primary cutaneous mucormycosis produced by the new species *Apophysomyces mexicanus*. *J. Clin. Microbiol.* **2014**, *52*, 4428-31.

44. Boodman, C.; Cheng, M.P. Nosocomial mucormycosis of the thigh. *I.D. Cases* **2021**, *25*, e01225.
45. Bowles, R.J.; Mitchell, J.J.; Price, C.; Ipaktchi, K. Severe mycosis as a rare infection after a corn auger injury of the hand: a case report. *Patient. Saf. Surg.* **2015**, *9*, 1-5.
46. Boyce, J.M.; Lawson, L.A.; Lockwood, W.R.; Hughes, J.L. *Cunninghamella bertholletiae* wound infection of probable nosocomial origin. *South Medical J*. **1981**, *74*, 1132-5.
47. Boyd, A.S.; Wiser, B.; Sams, H.H.; King Jr, L.E. Gangrenous cutaneous mucormycosis in a child with a solid organ transplant: a case report and review of the literature. *Pediatr. Dermatol*. **2003**, *20*, 411-5.
48. Buchta, V.; Kalous, P.; Otčenášek, M.; Váňová, M. Primary cutaneous *Absidia corymbifera* infection in a premature newborn. *Infection* **2003**, *31*, 57-9.
49. Burrell, S.; Ostlie, D.J.; Saubolle, M.; Dimler, M.; Barbour, S.D. *Apophysomyces elegans* infection associated with cactus spine injury in an immunocompetent pediatric patient. *Pediatr. Infect. Dis. J.* **1998**, *17*,663-4
50. Buzacott, K.; Townell, N.; Dettrick, A.; Grimwood, K. *Saksenaeae* subcutaneous abscess in an immunocompetent child. *Pediatr. Infect. Dis.* **2016**, *35*, 120.
51. Cáceres, A.M.; Sardiñas, C.; Marcano, C.; Guevara, R.; Barros, J.; Bianchi, G.; *et al*. *Apophysomyces elegans* limb infection with a favorable outcome: case report and review. *Clin. Infect. Dis*. **1997**, *25*, 331-2.
52. Camargo, J.F.; Yakoub, D.; Cho-Vega, J.H. Successful treatment of primary cutaneous mucormycosis complicating anti-TNF therapy with a combination of surgical debridement and oral posaconazole. *Mycopathologia* **2015**, *180,* 187-92.

53. Cantatore-Francis, J.L.; Shin, HT.; Heilman, E.; Glick, S.A. Primary cutaneous zygomycosis in two immunocompromised children. *Pediatr. Dermatol.* **2007**, *24*, 257-62.

54. Carceller, F.; Oñoro, G.; Buitrago, M.J.; Herrero, B.; Lassaletta, Á.; Pérez-Martínez, A.; *et al*. *Cunninghamella bertholletiae* infection in children: review and report of 2 cases with disseminated infection. *Pediatr. Hematol. Oncol*. **2014**, *36*, e109-14.

55. Carpenter, C.F.; Subramanian, A.K. *Cutaneous zygomycosis* (*mucormycosis*). *N. Engl. J. Med.* **1999**, *341*, 1891.

56. Carter, J.E.; Ulusarac, O. Widespread cutaneous involvement by invasive *Apophysomyces elegans* in a gravid patient following trauma. *Cutis* **2003**, *72,* 221-8.

57. Cataño, J.C.; Ramirez, I.C. Disseminated *Cunninghamella bertholletiae* infection. *Am. J. Med. Sci.* **2020**, *360*, e9-10.

58. Cateau, E.; Randriamalala, E.; Elsendoorn, A.; Giot, J.P.; Du Sorbier, C.M.; Rodier, M.H. Fatal-mixed cutaneous zygomycosis–aspergillosis: a case report. *Mycopathologia*. **2013**, *176*, 423-7.

59. Cefai, C.; Elliott, T.S.; Nutton, R.W.; Lockett, A.E.; Pooley, J. Zygomycotic gangrenous cellulitis. *Lancet* **1987**, *330*, 1337-8.

60. Chahal, H.S.; Abgaryan, N.; Lakshminarayanan, R.; Glover, A.T. Orbital mucormycosis following periorbital cutaneous infection. *Ophthalmic. Plast. Reconstr. Surg*. **2017**, *33*, S146-8.

61. Chakrabarti, A.; Kumar, P.; Padhye, A.A.; Chatha, L.; Singh, S.K.; Das, A.; *et al*. Primary cutaneous zygomycosis due to *Saksenaea vasiformis* and *Apophysomyces elegans*. *Clin. Infect. Dis*. **1997**, *24*, 580-2.

62. Chakrabarti, A.; Ghosh, A.; Prasad, G.S.; David, JK.; Gupta, S.; Das, A.; *et al*. *Apophysomyces elegans*: an emerging zygomycete in India. *J. Clin. Microbiol*. **2003**, *41*, 783-8.

63. Chakravarti, A.; Bhargava, R.; Bhattacharya, S. Cutaneous mucormycosis of nose and facial region in children: A case series. *Int. J. Pediatr. Otorhinolaryngol*. **2013**, *77*, 869-72.

64. Chambers, C.J.; Merin, M.R.; Fung, M.A.; Huntley, A.; Sharon, V.R. Primary cutaneous mucormycosis at sites of insulin injection. *J. Am. Acad. Dermatol*. **2011**, *64*, e79-81.

65. Chan, Y.; Goldwater, P.; Saxon, B. Successful treatment of cutaneous and subcutaneous zygomycosis in an immunosuppressed patient with aplastic anaemia. J. Paediatr. *Child Health* **2007**, *43*, 87-9.

66. Chander, J.; Kaur, M.; Bhalla, M.; Punia, R.S.; Singla, N.; Bhola, K.; Alastruey-Izquierdo, A.; Stchigel, A.M.; Guarro, J. Changing epidemiology of mucoralean fungi: chronic cutaneous infection caused by *Mucor irregularis*. *Mycopathologia* **2015**, *180*, 181-6.

67. Chander, J.; Kaur, J.; Attri, A.; Mohan, H. Primary cutaneous zygomycosis from a tertiary care centre in north-west India. Indian *J. Med. Res*. **2010**, *131*, 765-70.

68. Chander, J.; Stchigel, A.M.; Alastruey-Izquierdo, A.; Jayant, M.; Bala, K.; Rani, H.; *et al*. Fungal necrotizing fasciitis, an emerging infectious disease caused by *Apophysomyces* (Mucorales). *Rev. Iberoam. Micol*. **2015**, *32*, 93-8.

69. Chander, J.; Singla, N.; Kaur, M.; Punia, R.S.; Attri, A.; Alastruey-Izquierdo, A.; *et al*. *Saksenaea erythrospora*, an emerging mucoralean fungus causing severe necrotizing skin and soft tissue infections–a study from a tertiary care hospital in north India. *Infect. Dis*. **2016**, *49*, 170-7.

Articles included in the analysis for the systematic review

70. Chandler, F.W.; Watts, J.C.; Kaplan, W.; Hendry, A.T.; Mcginnis, M.R.; Ajello, L. Zygomycosis: report of four cases with formation of chlamydoconidia in tissue. *Am. J. Clin. Pathol.* **1985**, *84(1),* 99-103.
71. Chandra, S.; Woodgyer, A. Primary cutaneous zygomycosis due to *Mucor circinelloides.* Australas. *J. Dermatol*. **2002**, *43*, 39-42.
72. Chaney, S.; Gopalan, R.; Berggren, R.E. Pulmonary *Pseudallescheria boydii* infection with cutaneous zygomycosis after near drowning. *South Med. J*. **2004**, *97*, 683-8.
73. Chang, H.; Wang, P.; Huang, Y. Cutaneous mucormycosis. *Infection* **2018**, *46*, 901-902
74. Chawla, R.; Sehgal, S.; Kumar, S.R.; Mishra, B. A rare case of mucormycosis of median sternotomy wound caused by *Rhizopus arrhizus. Indian J. Med. Microbiol.* **2007***, 25,* 419-21.
75. Chen, Y.S.; Chen, C.L.; Liu, P.P.; Chiang, Y.C.; Sun, C.K.; Eng, H.L. Successful treatment of invasive mucormycosis following liver transplantation. *Transplant. Proc.* **1996**, *28,* 1708-9.
76. Cheng, V.C.; Chen, J.H.; Wong, SC.; Leung, S.S.; So, SY.; Lung, DC.; *et al*. Hospital outbreak of pulmonary and cutaneous zygomycosis due to contaminated linen items from substandard laundry. *Clin. Infect. Dis*. **2016**, *62*, 714-21.
77. Cheng, W.; Wang, G.; Yang, M.; Sun, L.; Dong, H.; Chen, Y.; Cheng, H. Cutaneous mucormycosis in a patient with lupus nephritis: A case report and review of literature. *Medicine* **2017**, *96*, e8211.
78. Chew, H.H.; Abuzeid, A.; Singh, D.; Tai, C.C. Surgical wound mucormycosis necessitating hand amputation: a case report. *J. Orthop. Surg*. **2008**, *16*, 267-9.
79. Clark, R.; Greer, D.L.; Carlisle, T.; Carroll, B. Cutaneous zygomycosis in a diabetic HTLV-I–seropositive man. *J. Am. Acad. Dermatol*. **1990**, *22*, 956-9.

80. Clark, F.L.; Batra, R.S.; Gladstone, H.B. Mohs micrographic surgery as an alternative treatment method for cutaneous mucormycosis. *Dermatol. Surg.* **2003***, 29*, 882-5.
81. Clauss, H.; Samuel, R. Simultaneous mold infections in an orthotopic heart transplant recipient. *Transpl. Infect. Dis.* **2008***, 10*, 343-5.
82. Cloughley, R.; Kelehan, J.; Corbett-Feeney, G.; Murray, M.; Callaghan, J.; Regan, P.; Cormican M. Soft tissue infection with *Absidia corymbifera* in a patient with idiopathic aplastic anemia. *J. Clin. Microbiol.* **2002***, 40*, 725-7.
83. Coerdt, K.M.; Zolper, E.G.; Starr, A.G.; Fan, K.L.; Attinger, C.E.; Evans, K.K. Cutaneous mucormycosis of the lower extremity leading amputation in two diabetic patients. *Arch. Plast. Surg.* **2021***, 48*, 231-6.
84. Cohen-Ludmann, C.; Kerob, D.; Feuilhade, M.; Chaine, B.; Guermazi, A.; Janier, M.; *et al*. Zygomycosis of the penis due to *Rhizopus oryzae* successfully treated with surgical debridement and a combination of high-dose liposomal and topical amphotericin B. *Arch. Dermatol.* **2006***, 142*, 1657-8.
85. Coleman, E.L.; Levy, L.; Panse, G.; Leventhal, J.S. Necrotic cheek ulcer in a liver transplant patient. *Int. J. Dermatol.* **2019***, 58*, 285-7.
86. Constantinides, J.; Misra, A.; Nassab, R.; Wilson, Y. *Absidia corymbifera* fungal infection in burns: a case report and review of the literature. *J. Burn Care Res.* **2008***, 29*, 416-9.
87. Cooter, R.D.; Lim, I.S.; Ellis, D.H.; Leitch, I.O. Burn wound zygomycosis caused by *Apophysomyces elegans*. *J. Clin. Microbiol.* **1990***, 28,* 2151-3.
88. Coronel-Pérez, I.M.; Rodríguez-Rey, E.M.; Castilla-Guerra, L.; Dominguez MC. Primary cutaneous mucormycosis due to *Saksenaea vasiformis* in an immunocompetent patient. *Actas dermosifiliogr.* **2015***, 106*, 516-8.

89. Corti, G.; Mondanelli, N.; Losco, M.; Bartolini, L.; Fontanelli, A.; Paradisi, F. Post-traumatic infection of the lower limb caused by rare Enterobacteriaceae and Mucorales in a young healthy male. *Int. J. Infect. Dis.* **2009***, 13*, e57-60.
90. Costa, A.R.; Porto, E.; Tayah, M.; Valente, N.Y.; Maranhão, W.M.; Rodrigues, M.C. Subcutaneous mucormycosis caused by *Mucor hiemalis* Wehmer f. *luteus* (Linnemann) Schipper 1973. *Mycoses.* **1990***, 33*, 241-6.
91. Craig, N.M.; Lueder, F.L.; Pensler, J.M.; Bean, B.S.; Petrick, M.L.; Thompson, R.B.; Eramo, L.R. Disseminated *Rhizopus* infection in a premature infant. *Pediatr. Dermatol.* **1994***, 11*, 346-50.
92. Dai, Y.; Walker, J.W.; Halloush, R.A.; Khasawneh, F.A. Mucormycosis in two community hospitals and the role of infectious disease consultation: a case series. *Int. J. Gen. Med*. **2013**, *6*, 833-8.
93. Darrisaw, L.; Hanson, G.; Vesole, D.H.; Kehl, S.C. *Cunninghamella infection* post bone marrow transplant: case report and review of the literature. *Bone Marrow Transplant.* **2000**, 25, 1213-6.
94. Dave, S.P.; Vivero, R.J.; Roy, S. Facial cutaneous mucormycosis in a full-term infant. Arch. Otolaryngol. *Head Neck Surg.* **2008***, 134*, 206-9.
95. Davidson, N.; Campbell, K.; Foroughi, F.; Tayal, V.; Lynar, S.; Crawford, L.C.; *et al*. Disseminated *Saksenaea* infection in an immunocompromised host associated with a good clinical outcome: a case report and review of the literature. *BMC Infect. Dis.* **2020***, 20*, 1-5.
96. Davuodi, S.; Manshadi, S.A.; Salehi, M.R.; Yazdi, F.; Khazravi, M.; Fazli, J.T. Fatal cutaneous mucormycosis after kidney transplant. *Exp. Clin. Transplant.* **2015***, 13*, 82-5.

97. Dayal, D.; Jain, P.; Kumar, R.; Bakshi, J.; Menon, P.; Das, A.; *et al*. Clinical spectrum and outcome of invasive filamentous fungal infections in children with Type 1 diabetes: North Indian experience. *Clin. Pediatr. Endocrinol.* **2015***, 24*, 51-7.
98. De Chaumont, A.; Pierret, C.; Janvier, F.; Goudard, Y.; de Kerangal, X.; Chapuis, O. Mucormycosis: a rare complication of an amputation. *Ann. Vasc. Surg.* **2014***, 28*, 1035.e15-9.
99. De Decker, K.; Van Poucke, S.; Wojciechowski, M.; Ieven, M.; Colpaert, C.; Vogelaers, D.; Jorens P.G. Successful use of posaconazole in a pediatric case of fungal necrotizing fasciitis. *Pediatr. Crit. Care Med.* **2006***, 7*, 482-5.
100. De Queiroz Telles Filho, F.; Coelho, A.; Porto, E.; Lameira, R.F.; Freitas, M.M.; Barboza J.; Ramos J.L. Subcutaneous mucormycosis caused by Rhizopus oryzae: probable nosocomial acquired infection. *Rev. Inst. Med. Trop. São Paulo* **1985***, 27*, 201-6.
101. Dean, D.F.; Ajello, L.; Irwin, R.S.; Woelk, W.K.; Skarulis, G.J. Cranial zygomycosis caused by *Saksenaea vasiformis*: case report. *J. Neurosurg.* **1977***, 46*, 97-103.
102. Dela Cruz, W.P.; Calvano, T.P.; Griffith, M.E.; White, C.E.; Kim, S.H.; Sutton, D.A.; *et al*. Invasive *Apophysomyces variabilis* infection in a burn patient. *J. Clin. Microbiol.* **2012***, 50*, 2814-7.
103. Delie, A.; Vlummens, P.; Creytens, D.; Steel, E. Cutaneous mucormycosis as result of insulin administration in an AML patient: Case report and review of the literature. *Acta Clin. Belg.* **2016***, 72*, 352-6.
104. Dennis, J.E.; Rhodes, K.H.; Cooney, D.R.; Roberts, G.D. Nosocomial *Rhizopus* infection (zygomycosis) in children. *J. Pediatr.* **1980***, 96*, 824-8.

105. Desai, R.P.; Joseph, N.M.; Ananthakrishnan, N.; Ambujam, S. Subcutaneous zygomycosis caused by *Mucor hiemalis* in an immunocompetent patient. *Australas. Med. J.* **2013***, 6*, 374-7.

106. Devi, S.C.; Kanungo, R.; Barreto, E.; Thomas, A.G.; Shashikala, N.; Srinivasan, S.; Anitha, P.K. Favorable outcome of amphotericin B treatment of zygomycotic necrotizing fascitis caused by *Apophysomyces elegans*. *Int. J. Dermatol.* **2008***, 47*, 407-9.

107. Di Pentima, M.C.; Chan, S.; Powell, J.; Napoli, J.A.; Walter, A.W.; Walsh, T.J. Topical amphotericin B in combination with standard therapy for severe necrotizing skin and soft-tissue mucormycosis in an infant with bilineal leukemia: case report and review. *J. Pediatr. Hematol. Oncol.* **2014***, 36,* e468-70.

108. Diamond, H.J.; Phelps, R.G.; Gordon, M.L.; Lambroza, E.; Namdari, H.; Bottone, E.J. Combined Aspergillus and zygomycotic (*Rhizopus*) infection in a patient with acquired immunodeficiency syndrome: presentation as inflammatory tinea capitis. *J. Am. Acad. Dermatol.* **1992***, 26*, 1017-8.

109. Dickinson, M.; Kalayanamit, T.; Yang, CA.; Pomper, GJ.; Franco-Webb, C.; Rodman D. Cutaneous zygomycosis (mucormycosis) complicating endotracheal intubation: diagnosis and successful treatment. *Chest* **1998***, 114*, 340-2.

110. Ding, X.L.; Li, H.M.; Du, J. Successful treatment of cutaneous mucormycosis disseminated from pulmonary mucormycosis with liposomal amphotericin B and posaconazole. *Chin. Med. J.* **2020**, 133, 1747-8.

111. Dizbay, M.; Adisen, E.; Kustimur, S.; Sari, N.; Cengiz, B.; Yalcin, B.; *et al*. Fungemia and cutaneous zygomycosis due to Mucor circinelloides in an intensive care unit patient: case report and review of literature. *Jpn. J. Infect. Dis.* **2009***, 62*, 146-8.

112. Dodémont, M.; Hites, M.; Bailly, B.; Trepant, A.L.; De Mendonça, R.; Denis, O.; *et al*. When you can't see the wood for the trees. *Mucor circinelloides*: a rare case of primary cutaneous zygomycosis. *J. Mycol. Med.* **2015***, 25*, 151-4.

113. Downie, M.L.; Alghounaim, M.; Davidge, K.M.; Yau, Y, Walsh, T.J.; Pope, E.; *et al*. Isolated cutaneous mucormycosis in a pediatric renal transplant recipient. *Pediatr. Transplant.* **2018***, 22*, e13172.

114. Duffy, J.; Harris, J.; Gade, L.; Sehulster, L.; Newhouse, E.; O'Connell, H.; Noble-Wang, J.; Rao, C, Balajee, S.A.; Chiller, T. Mucormycosis outbreak associated with hospital linens. *Pediatr. Infect. Dis. J.* ***2014****, 33*, 472-6.

115. Du Plessis, P.J.; Wentzel, L.F.; Delport, S.D.; Van Damme, E. Zygomycotic necrotizing cellulitis in a premature infant. *Dermatology.* **1997***, 195*, 179-81.

116. Durand, C.M.; Alonso, C.D.; Subhawong, A.P.; Kwiatkowski, N.P.; Showel, M.; Carroll, K.C.; Marr, K.A. Rapidly progressive cutaneous *Rhizopus microsporus* infection presenting as Fournier's gangrene in a patient with acute myelogenous leukemia. *Transpl. Infect. Dis.* **2011***, 13*, 392-6.

117. Eaton, M.E.; Padhye, A.A.; Schwartz, D.A.; Steinberg, J.P. Osteomyelitis of the sternum caused by *Apophysomyces elegans*. *J. Clin. Microbiol.* **1994***, 32*, 2827-8.

118. Echaiz, J.F.; Burnham, C.A.; Bailey, T.C. A case of *Apophysomyces trapeziformis* necrotizing soft tissue infection. *Int. J. Infect. Dis.* **2013***, 17*, e1240-2.

119. Egge, S.; Wei, E.; Clements, E.; Chandranesan, A.S. Post-traumatic fatal disseminated Apophysomyces elegans infection. *Med. Mycol. Case Rep.* **2018***, 22*, 45-7.

120. El Deeb, Y.; Al Soub, H.; Almaslamani, M.; Al Khuwaiter, J.; Taj-Aldeen, S.J. Post-traumatic cutaneous mucormycosis in an immunocompetent patient. *Ann. Saudi Med.* **2005***, 25*, 343-5.

121. Elsiesy, H.; Saad, M.; Shorman, M.; Amr, S.; Abaalkhail, F.; Hashim, A.; *et al*. Invasive mucormycosis in a patient with liver cirrhosis: case report and review of the literature. *Hepat. Mon.* **2013**, *13*, e10858.

122. Elzein, F.; Mohammed, N.; Arafah, M.; Albarrag, A.; Habib, R.; Faqehi, A. Complication of massive trauma by fungal infection and bone tuberculosis. *Med. Mycol. Case Rep.* **2019**, *27*, 4-7.

123. Elzein, F.; Albarrag, A.; Kalam, K.; Arafah, M.; Al-Baadani, A.; Eltayeb, N.; *et al*. Mucormycosis: An 8-year experience of a tertiary care centre in Saudi Arabia. *J. Infect. Public Health* **2020**, *13*, 1774-9.

124. El Zein, S.; El-Sheikh, J.; Zakhem, A.; Ibrahim, D.; Bazarbachi, A.; Kanj, S.S. Mucormycosis in hospitalized patients at a tertiary care center in Lebanon: a case series. *Infection* **2018**, *46*, 811-21.

125. Everett, E.D.; Pearson, S.; Rogers, W. *Rhizopus* surgical wound infection associated with elasticized adhesive tape dressings. *Arch. Surg.* **1979**, *114*, 738-9.

126. Fernández Tormos, E.; Corella Montoya, F.; Martínez Izquierdo, M.Á.; Sánchez-Artola, B.; Limousin Aranzabal, B.; Larraínzar-Garijo, R. Infection due to *Saksenaea vasiformis* following a spider bite. *J. Hand Surg. Am.* **2019**, *44*, 619.e1-619.e5.

127. Fingeroth J.D.; Roth R.S.; Talcott J.A.; Rinaldi M.G. Zygomycosis due to *Mucor circinelloides* in a neutropenic patient receiving chemotherapy for acute myelogenous leukemia. *Clin. Infect. Dis.* **1994**, *19*, 135-7.

128. Fisher, J.; Tuazon, C. U.; Geelhoed, G.W. Mucormycosis in transplant patients. *Am. Surg.* **1980**, *46*, 315-22.

129. Fu, M.H.; Liu, J.; Liang, G.Z.; Li, CR.; Zhu, X.M, Wang, L.; *et al*. Successful treatment of eczema-like mucormycosis in a child by combination of intravenous drip and percutaneous injection amphotericin B. *Mycopathologia* **2019***, 184*, 309-13.

130. Fujimoto, A.; Nagao, K.; Tanaka, K.; Yamagami, J.; Udagawa, S.I.; Sugiura, M. The first case of cutaneous mucormycosis caused by *Rhizopus azygosporus*. *Br. J. Dermatol.* **2005***, 153,* 428-30.

131. Gadadhar, H.; Hawkins, S.; Huffstutter, J.E.; Panda, M. Cutaneous mucormycosis complicating methotrexate, prednisone, and infliximab therapy. *J. Clin. Rheumatol.* **2007***, 13*, 361-2.

132. Garbino, J.; Uçkay, I.; Amini, K.; Puppo, M.; Richter, M.; Lew, D. *Absidia* posttraumatic infection: successful treatment with posaconazole. *J. Infect.* **2005***, 51*, e135-8.

133. García-Bustínduy, M.; Guimerá-Martín-Neda, F.; Noda, A.; Lecuona, M.; Sánchez-González, R.; González de Mesa, M.J.; *et al*. Primary cutaneous mucormycosis: a diagnosis to consider. *J. Eur. Acad. Dermatol. Venereol.* **1999***, 12*, 258-62.

134. García-Pajares, F.; Sánchez-Antolín, G.; Almohalla Alvárez, C.; Madrigal Rubiales, B.; Núñez-Rodríguez, H.; Sancho del Val, L.; *et al*. Cutaneous mucormycosis infection by Absidia in two consecutive liver transplant patients. *Transplant. Proc.* **2012***, 44*, 1562-4.

135. García-Sepúlveda, R.; Navarrete-Solís, J.; Villanueva-Lozano, H.; de J Treviño-Rangel R.; González, GM.; Enríquez-Rojas, J.; *et al*. Photoletter to the editor: Atypical primary cutaneous mucormycosis of the scalp. *J. Dermatol. Case Rep.* **2017***, 11*, 32- 4.

136. Gardiner, BJ.; Simpson, I.; Khuu, MH.; Kidd, SE.; Lo, CH.; Jenkin, GA. An unusual ulcer: A case of cutaneous mucormycosis caused by *Rhizopus oryzae*. *Med. Mycol. Case Rep.* **2014***, 7*, 8-11.

137. Garg, J.; Sujatha, S.; Garg, A.; Parija, S.C. Nosocomial cutaneous zygomycosis in a patient with diabetic ketoacidosis. *Int. J. Infect. Dis.* **2009***, 13*, e508-10.

138. Garrido, P.M.; Pimenta, R.; Viana, I.; Kutzner, H.; Filipe, P.; Soares-Almeida, L. Cutaneous mucormycosis mimicking pancreatic panniculitis. *J. Cutan. Pathol.* **2020***, 48*, 1007-9.

139. Gartenberg, G.; Bottone, E.J.; Keusch, G.T, Weitzman, I. Hospital-acquired mucormycosis (*Rhizopus rhizopodiformis*) of skin and subcutaneous tissue: epidemiology, mycology and treatment. *N. Engl. J. Med.* **1978***, 299*, 1115-8.

140. Geisen, M.; Fodor, P.; Eich, G.; Zollinger, A.; Dzemali, O.; Blumenthal, S. Disseminated cutaneous mucormycosis in a patient on high-dose steroid therapy for severe ARDS. *Intensive Care Med.* **2011***, 37*, 1895-6.

141. Geller JD.; Peters MS.; Su WP. Cutaneous mucormycosis resembling superficial granulomatous pyoderma in an immunocompetent host. *J. Am. Acad. Dermatol.* ***1993,*** *29*, 462-5.

142. Gelman, A.; Valdes-Rodriguez, R.; Bhattacharyya, S.; Yosipovitch, G. A case of primary cutaneous mucormycosis caused by minor trauma. *Dermatol. Online. J.* **2015***, 21*, 13030/qt8qn2g3zv.

143. Ghaderkhani, S.; Ahmadinejad, Z.; Dashti, H.; Safaei, M.; Ghiasvand, F. Wound infection with an unusual pathogen after liver transplantation. *Case Rep. Transplant.* **2020***, 2020*, 8396507.

144. Ghaemi, N.; Bagheri, S.; Shirdelzade, S. Pulmonary and cutaneous mucormycosis in two children with diabetes mellitus type 1. *J. Pediatr. Endocrinol. Metab.* **2021***, 34,* 941-5.

145. Giordano, G.N.; Abiega, C.; Chipre, E.; Garcia-Effron, G.; Caeiro, J.P. Severe *Cunninghamella* infection in an HIV patient: After cenotes exposure in Mexico. *Open J. Clin. Med. Case Rep.* **2019***, 5*, 1545.

146. Giuliani, A.; Mettimano, M.; Viviani, D.; Scagliusi, A.; Bruno, A.; Russo, A.; *et al*. An uncommon case of systemic mucormycosis associated with spinal cord infarction in a recently diagnosed diabetic. *Int. J. Immunopathol. Pharmacol.* **2010***, 23*, 355-8.

147. Gkegkes, I.D.; Kotrogiannis, I.; Konstantara, F.; Karetsou, A.; Tsiplakou, S.; Fotiou, E.; *et al*. Cutaneous mucormycosis by *Saksenaea vasiformis*: an unusual case report and review of literature. *Mycopathologia* **2019***, 184*, 159-167.

148. Goldschmied-Reouven, A.; Shvoron, A.; Topaz, M.; Block, C. *Saksenaea vasiformis* infection in a burn wound. *J. Med. Vet. Mycol.* **1989***, 27*, 427-9.

149. Gómez-Camarasa, C.; Rojo-Martín, M.D.; Miranda-Casas, C.; Alastruey-Izquierdo, A.; Aliaga-Martínez, L.; Labrador-Molina, J.M.; Navarro-Marí, J.M. Disseminated infection due to Saksenaea vasiformis secondary to cutaneous mucormycosis. *Mycopathologia* **2014***, 177*, 97-101.

150. González-Abad, M.J.; Alonso Sanz, M. Zygomycosis in children: disseminated infection caused by *Cunninghamella bertholletiae*. *Arch. Bronconeumol.* **2013***, 49*, 35.

151. Gordon, G.; Indeck, M.; Bross, J.; Kapoor, D.A.; Brotman, S. Injury from silage wagon accident complicated by mucormycosis. *J. Trauma* **1988***, 28*, 866-7.

152. Gowdara, V.K.; Shivappa, S.G.; Puttaswamy, M.C.; Ramakrishna, R.; Ramalingaiah, R.; Puranik, V. *Apophysomyces variabilis* a flesh eating fungus – a case report. *I.OS.R.-J. Dental Med. Sci.* **2014***, 13*, 86-90.

153. Goyal, A.; Tyagi, I.; Syal, R.; Marak, R.S.; Singh, J. *Apophysomyces elegans* causing acute otogenic cervicofacial zygomycosis involving salivary glands. *Med. Mycol.* **2007***, 45*, 457-61.

154. Grossklaus, D.J.; Dutta, S.C.; Shappel, S.; Kirchner F.K. Cutaneous mucormycosis presenting as a penile lesion in a patient with acute myeloblastic leukemia. *J. Urol.* **1999***, 161*, 1906-7.

155. Guarro, J.; Chander, J.; Alvarez, E.; Stchigel, A.M.; Robin, K.; Dalal, U.; *et al*. *Apophysomyces variabilis* infections in humans. *Emerg. Infect. Dis.* **2011***, 17*, 134-5.

156. Gupta, A.; Jain, S.; Agrawal, C.; Kapoor, G. Successful outcome of mucormycosis in two children on induction therapy for acute lymphoblastic leukemia. *Indian. J. Med. Paediatr. Oncol.* **2013***, 34*, 313-6.

157. Hackenberg, T.A.; Evans.; T.G. Localized cutaneous Rhizopus presenting as an anesthetic rash in a patient with human immunodeficiency virus. *Clin. Infect. Dis*. **1993**, *17*, 949.

158. Hadaschik, E.; Koschny, R.; Willinger, B.; Hallscheidt, P.; Enk, A.; Hartschuh, W. Pulmonary, rhino-orbital and cutaneous mucormycosis caused by *Rhizomucor pusillus* in an immunocompromised patient. *Clin. Exp. Dermatol.* **2012***, 37*, 355-7.

159. Hadithi, M.; Leemans, CR.; Jonkhoff, AR. The irreplaceable image: Cutaneous mucormycosis in acute myeloid leukemia; hit hard and quickly. *Haematologica* **2001***, 86*, 1320.

160. Hall, J.C.; Brewer.; J.H.; Reed, W.A.; Steinhaus, D.M.; Watson, K.R. Cutaneous mucormycosis in a heart transplant patient. *Cutis* **1988**, 42, 183-6.

161. Hammond, D.E.; Winkelmann, R.K. Cutaneous phycomycosis. Report of three cases with identification of Rhizopus. *Arch. Dermatol.* **1979***, 115*, 990-2.

162. Hampson, F.G.; Ridgway, E.J.; Feeley, K.; Reilly, J.T. A fatal case of disseminated zygomycosis associated with the use of blood glucose self-monitoring equipment. *J. Infect.* **2005***, 51*, e269-72.

163. Hansen, J.P.; Kristjansson, A.K.; Stone, M.S. A neutropenic child with a purple lesion on the thigh. *Pediatr. Dermatol.* **2007***, 24,* 560-1.

164. Haque, H.; Nettboy, S.; Kumar, S. Surgical-site mucormycosis infection in a solid-organ transplant recipient and a concise review of the literature. *B.M.J. Case Rep.* **2019***, 12*, e229687.

165. Harada, A.S.; Lau, W. Successful treatment and limb salvage of mucor necrotizing fasciitis after kidney transplantation with posaconazole. *Hawaii Med. J*. **2007**, *66*, 68-71.

166. Harman, M.; Uçmak, D.; Dal, T. A rare case of mucormycosis in the scalp. *Acta Med. Port.* **2013***, 26*, 754-7.

167. Hata, T.R.; Johnson, R.A.; Barnhill, R.; Dover, J.S. Ecthymalike lesions on the leg of an immunocompromised patient. Primary cutaneous mucormycosis. *Arch. Dermatol.* **1995***, 131*, 833-4, 836-7.

168. Hawkes, J.E.; Florell, S.R.; Wada, D.A. Chronic, painful, nonhealing ulcer on the right arm following minor trauma. *J.A.M.A. Dermatol.* **2015***, 151*, 787-8.

169. Hay, R.J.; Campbell, C.K.; Marshall, W.M.; Rees, B.I.; Pincott, J. Disseminated zygomycosis (mucormycosis) caused by *Saksenaea vasiformis*. *J. Infect.* **1983***, 7,* 162-5.

170. Heinz, T.; Perfect, J.; Schell, W.; Ritter, E.; Ruff, G.; Serafin, D. Soft-tissue fungal infections: surgical management of 12 immunocompromised patients. *Plast. Reconstr. Surg.* **1996**, *97*, 1391-9.

171. Hemashettar, B.M.; Patil, R.N.; O'Donnell, K.; Chaturvedi, V.; Ren, P.; Padhye, A.A. Chronic rhinofacial mucormycosis caused by *Mucor irregularis* (*Rhizomucor variabilis*) in India. *J. Clin. Microbiol.* **2011**, *49*, 2372-5.

172. Heydari, A.A.; Fata, A.; Mojtabavi, M. Chronic cutaneus mucormycosis in an immunocompetent female. *Iran Red Crescent Med. J.* **2013**, *15*, 254-5.

173. Hicks, W.L Jr.; Nowels, K.; Troxel, J. Primary cutaneous mucormycosis. *Am. J. Otolaryngol.* **1995**, *16*, 265-8.

174. Hocker, T.L.; Wada, D.A.; Bridges, A.; el-Azhary, R. Disseminated zygomycosis heralded by a subtle cutaneous finding. *Dermatol. Online J*. **2010**, *16*, 3.

175. Hopwood, V.; Hicks, D.A.; Thomas, S.; Evans, E.G. Primary cutaneous zygomycosis due to *Absidia corymbifera* in a patient with AIDS. *J. Med. Vet. Mycol.* **1992**, *30*, 399-402.

176. Horré, R.; Jovanić.; B, Herff, S.; Marklein.; G, Zhou.; H, Heinze, I.; *et al*. Wound infection due to *Absidia corymbifera* and *Candida albicans* with fatal outcome. *Med. Mycol.* **2004**, *42*, 373-8.

177. Hospenthal, D.R.; Chung, K.K.; Lairet, K.; Thompson, E.H.; Guarro, J.; Renz, E.M.; Sutton, D.A. *Saksenaea erythrospora* infection following combat trauma. *J. Clin. Microbiol.* **2011**, *49*, 3707-9.

178. Hoyos, Á.; Mejía, M.A.; Herrera, V.; Soto, A.; Rico, C. Necrotizing cellulitis due to *Rhizopus arrhizus* in an extremely premature infant. *I.D. Cases* **2020**, *20*, e00754.

179. Hsieh, T.T.; Tseng, H.K.; Sun, P.L.; Wu, Y.H.; Chen, G.S. Disseminated zygomycosis caused by *Cunninghamella bertholletiae* in patient with hematological malignancy and review of published case reports. *Mycopathologia* **2013***, 175*, 99-106.

180. Huang, Y.T.; Liao, C.H.; Hsueh, P.R. Image Gallery: Cutaneous infections caused by *Alternaria alternata* and *Mucor irregularis* 1 year apart in a patient with iatrogenic Cushing syndrome. *Br. J. Dermatol.* **2016***, 174(6),* e82.

181. Huffnagle, K.E.; Southern, P.M Jr.; Byrd, L.T.; Gander, R.M. *Apophysomyces elegans* as an agent of zygomycosis in a patient following trauma. *J. Med. Vet. Mycol.* **1992***, 30*, 83-6.

182. Hughes, C.; Driver, S.J.; Alexander, K.A. Successful treatment of abdominal wall Rhizopus necrotizing cellulitis in a preterm infant. *Pediatr. Infect. Dis. J.* **1995***, 14*, 336.

183. Humphrey, V.S.; Li, X.; Choudhary, S.; Patton, T. Fatal disseminated mucormycosis in a hematological immunocompromised patient with extensive voriconazole exposure: a case report and review of the literature. *Case Rep. Dermatol.* **2020***, 12*, 168-73.

184. Hunter A.J.; Bryant R.E. Abdominal wall mucormycosis successfully treated with amphotericin and itraconazole. *J. Infect.* **2002***, 44,* 203-4.

185. Hurlé A.; Campos-Herrero M.I.; Rodríguez H.; Elcuaz R.; Arroyo J.; Floriano P.; Abad C. Cutaneous mucormycosis of the thoracic wall. *Clin. Infect. Dis.* **1996***, 22*, 373-4.

186. Iida T.; Sawada N.; Takahashi M.; Zendejas I.R.; Kayler L.K.; Magliocca J.F.; *et al*. Successful treatment of invasive mucormycosis in a liver transplant patient by arm amputation. *Transplant. Proc.* **2010***, 42*, 2794-6.

187. Ilyas, S.; Al-Abbadi, M.A.; Raval, B.; Shams, W.E. Mucor causing nonhealing skin ulcer diagnosed by scrape cytology: description of unusual presentation. *Diagn. Cytopathol.* **2011**, *39*, 714-5.

188. Ingram, P.R.; Suthananthan, A.E.; Rajan, R.; Pryce, T.M.; Sieunarine, K.; Gardam, D.J.; Heath, C.H. Cutaneous mucormycosis and motor vehicle accidents: Findings from an Australian case series. *Med. Mycol.* **2014***, 52*, 819-25.

189. Iwen, P.C.; Sigler, L.; Noel, R.K.; Freifeld, A.G. *Mucor circinelloides* was identified by molecular methods as a cause of primary cutaneous zygomycosis. *J. Clin. Microbiol.* **2007***, 45*, 636-40.

190. Iyengar, S.; Chambers, C.J.; Millsop, J.W.; Fung, M.A.; Sharon, V.R. Purple patches in an immunocompromised patient: a report of secondary disseminated cutaneous mucormycosis in a man with chronic lymphocytic leukemia. *Dermatol. Online J.* **2017***, 23*, 13030/qt8cm6m764.

191. Jain, J.K.; Markowitz, A.; Khilanani, P.V.; Lauter, C.B. Localized mucormycosis following intramuscular corticosteroid. Case report and review of the literature. *Am. J. Med. Sci.* **1978***, 275*, 209-16.

192. Jain, S.K.; Kaza, R.C.; Tanwar, R. Mucormycosis of the anterior chest wall presenting as a soft tissue tumour. *J. Wound Care* **2011***, 20*, 176-8.

193. Jantunen, E.; Kolho, E.; Ruutu, P.; Koukila-Kähkölä, P.; Virolainen, M.; Junoven, E.; Volin, L. Invasive cutaneous mucormycosis caused by *Absidia corymbifera* after allogeneic bone marrow transplantation. *Bone Marrow Transplant*. **1996**, *18*, 229-30.

194. Jevalikar, G.; Sudhanshu, S.; Mahendru, S.; Sarma, S.; Farooqui, KJ.; Mithal, A. Cutaneous mucormycosis as a presenting feature of type 1 diabetes in a boy - case report and review of the literature. *J. Pediatr. Endocrinol. Metab.* **2018***, 31*, 689-92.

195. Jimenez-Cauhe, J.; Molins-Ruiz, M.; Fernandez-Guarino, M. Rapidly progressing ulcer and a urine drainage bag. *Dermatol. Online J.* **2018***, 24*, 13030/qt4r53q48g.

196. Jiménez, C.; Lumbreras, C.; Aguado, J.M.; Loinaz, C.; Paseiro, G.; Amado, A.; Morales, J.M.; Sánchez, G.; García, I.; del Palacio, A.; Moreno, E. Successful treatment of mucor infection after liver or pancreas-kidney transplantation. *Transplantation* **2002***, 73*, 476-80.

197. Jin, H.; Cho, H.H.; Kim, W.J.; Song, M.; Kim, H.S.; Ko, H.C.; *et al*. Cutaneous *Mucor hiemalis* infection diagnosed by molecular methods. *Int. J. Dermatol.* **2015***, 54*, e546-8.

198. Johnson, V.E.; Boyd, A.S. Primary cutaneous *Rhizopus* folliculitis in an immunocompetent woman. *J.A.A.D. Case Rep.* **2017***, 4*, 36-7.

199. Johnson, P.C.; Satterwhite, T.K.; Monheit, JE.; Parks D. Primary cutaneous mucormycosis in trauma patients. *J. Trauma* **1987***, 27*, 437-41.

200. Johnson, K.E.; Leahy, K.; Owens, C.; Blankson, J.N.; Merz, W.G.; Goldstein, B.J. An atypical case of fatal zygomycosis: simultaneous cutaneous and laryngeal infection in a patient with a non-neutropenic solid prostatic tumor. *Ear Nose Throat J.* **2008***, 87,* 152-5.

201. Jones, N.F.; Shin, E.K.; Eo, S, Starzl, T.E. Successful salvage of mucormycosis infection of the forearm and osteomyelitis of the ulna. *Hand (N. Y.)* **2008***, 3,* 332-6.

202. Jones, M.U.; Flores, M.S.; Vereen, R.J.; Szabo, S.R.; Logemann, N.F.; Eberly, M.D. Self-resolving superficial primary cutaneous mucormycosis in a 7-week-old infant. *Pediatr. Dermatol.* **2018***, 35*, e248-e250.

203. Josefiak, E.J.; Foushee, J.S.; Smith, L.C. Cutaneous mucormycosis. *Am. J. Clin. Pathol.* ***1958****, 30*, 547-52.

204. Jundt, J.S.; Wong, M.E.K.; Tatara, A.M.; Demian N.M. Invasive cutaneous facial Mucormycosis in a Trauma Patient. *J. Oral Maxillofac. Surg.* **2018***, 76*, 1930.e1-1930.e5.

205. Kamalam, A.; Thambiah, AS. Cutaneous infection by *Syncephalastrum*. *Sabouraudia* **1980***, 18,* 19-20.

206. Kanagaraju, V.; Narayanasamy, V.K.; Sukumaran, S.; Moorthy, U.; Sundar, V.S.; Lakshmi, S.V. Invasive, gangrenous mucormycosis of arm: A fatal opportunistic infection in a highly immunocompromised host. *Indian J. Pathol. Microbiol.* **2019***, 62*, 618-620.

207. Kanemaru, M.; Tashima, S.; Yamazaki.; A, Masuda, K.; Nagoshi.; H, Kobayashi, T.; *et al*. Disseminated mucormycosis due to *Rhizopus oryzae* diagnosed by skin biopsy. *J. Dermatol.* **2014***, 42*, 100-1.

208. Kang, D.; Jiang, X.; Wan, H.; Ran, Y.; Hao, D.; Zhang, C. *Mucor irregularis* infection around the inner canthus cured by amphotericin B: a case report and review of published literatures. *Mycopathologia* **2014***, 178*, 129-33.

209. Kapadia, S.; Polenakovik, H. Cutaneous zygomycosis following attempted radial artery cannulation. *Skinmed* **2004***, 3*, 336-8.

210. Karam, A.; Ianotto, J.C.; Metges, J.P.; Eveillard, J.R.; Leroy, J.P.; Le Flohic, A.M.; Berthou, C. Ulceration of the penis due to *Absidia corymbifera*. *Br. J. Dermatol.* **2003***, 148*, 1286-7.

211. Karim, M.; Ahmed, R.; Chishty, K. Wound zygomycosis: two cases with unusual manifestations. *Int. J. Infect. Dis.* **2001***, 5*, 107-11.

212. Kaur, R.; Bala, K.; Ahuja, R.B.; Srivastav, P.; Bansal, U. Primary cutaneous mucormycosis in a patient with burn wounds due to Lichtheimia ramose. *Mycopathologia* **2014**, *178*, 291-5.

213. Kaushik, R.; Chander, J.; Gupta, S.; Sharma, R.; Punia, R.S. Fatal primary cutaneous zygomycosis caused by Saksenaea vasiformis: case report and review of literature. *Surg. Infect. (Larchmt)* **2012***, 13*, 125-9.

214. Kawasaki, M.; Kawakami, N.; Kawai, K.; Kanekura, T. Cutaneous mucormycosis in bone marrow transplantation recipients. *Eur. J. Dermatol.* **2012***, 22*, 578-9.

215. Kefala-Agoropoulou, K.; Farmaki, E.; Tsiouris, J.; Roilides, E.; Velegraki, A. Cutaneous zygomycosis in an infant with Pearson syndrome. *Pediatr. Blood Cancer* **2008***, 50*, 939-40.

216. Kelpin, J.; Fahrenkopf, M.; Kelley, J.; Eichhorn, M.; Martin, M. Mucormycosis Osteomyelitis of the *Hand. J. Hand Surg. Am.* **2018***, 44*, 424.e1-4.

217. Kerbaul, F.; Guidon, C.; Collart, F.; Lépidi, H.; Cayatte, B.; Bonnet, M.; *et al*. Abdominal wall mucormycosis after heart transplantation. J*. Cardiothorac. Vasc. Anesth.* **2004***, 18*, 822-3.

218. Kerr, OA.; Bong, C.; Wallis, C.; Tidman, MJ. Primary cutaneous mucormycosis masquerading as pyoderma gangrenosum. *Br. J. Dermatol.* **2004***, 150*, 1212-3.

219. Khan, F.A.; Vinjamuri, M.; Sarwari, A. An immunocompromised patient with necrotic chin lesions. *Clin. Infect. Dis.* **2006***, 42*, 242-3, 296-7.

220. Khardori, N.; Hayat, S.; Rolston, K.; Bodey, G.P. Cutaneous *Rhizopus* and *Aspergillus* infections in five patients with cancer. *Arch. Dermatol.* **1989***, 125*, 952-6.

221. Khatri, A.; Chang, K.M.; Berlinrut, I.; Wallach F. Mucormycosis after Coronavirus disease 2019 infection in a heart transplant recipient - Case report and review of literature. *J. Mycol. Med.* **2021***, 31*, 101125.

222. Kimura, M.; Smith M.B.; McGinnis, M.R. Zygomycosis due to *Apophysomyces elegans*. Report of 2 cases and review of the literature. *Arch. Pathol. Lab. Med.* **1999***, 123*, 386-90.

223. Kindo, A.J.; Shams, N.R.; Kumar, K.; Kannan, S.; Vidya, S.; Kumar, A.R.; Kalyani, J. Fatal cellulitis caused by *Apophysomyces elegans. Indian. J. Med. Microbiol.* **2007***, 25*, 285-7.

224. Kobayashi M.; Hiruma M.; Matsushita A.; Kawai M.; Ogawa H.; Udagawa S. Cutaneous zygomycosis: a case report and review of Japanese reports. *Mycoses* **2001***, 44*, 311-5.

225. Kokkayil, P.; Pandey, M.; Agarwal, R.; Kale, P.; Singh, G.; Xess, I. *Rhizopus homothallicus* causing invasive infections: series of three cases from a single centre in North India. *Mycopathologia* **2017***, 182*, 921-926.

226. Koklu, E.; Akcakus, M.; Torun, Y.A.; Tulpar, S.; Tasdemir, A. Primary gangrenous cutaneous mucormycosis of the scalp in a child: a case report. *Pediatr. Emerg. Care* **2008***, 24,* 102-4.

227. Kompoti, M.; Michalia, M.; Kallitsi, G.; Giannopoulou, P.; Arabatzis, M.; Liapi, G.; *et al*. Fatal cutaneous *Saksenaea vasiformis* infection in a critically ill trauma patient. *Mycoses* **2011***, 54*, e599-601.

228. Konigsberg, M.W.; Wu, C.H.; Strauch, R.J. Topical treatment for cutaneous mucormycosis of the upper extremity. *J. Hand Surg. Am.* **2020***, 45*, 1189.e1-1189.e5.

229. Kontogiorgi, M.; Floros, I.; Koroneos, A.; Vamvouka, C.; Paniara, O.; Roussos, C.; Routsi, C. Fatal post-traumatic zygomycosis in an immunocompetent young patient. J. *Med. Microbiol.* **2007***, 56*, 1243-5.

230. Kordy, F.N.; Al-Mohsen, I.Z.; Hashem, F.; Almodovar, E.; Al Hajjar, S.; Walsh, T.J. Successful treatment of a child with posttraumatic necrotizing fasciitis caused by

Apophysomyces elegans: case report and review of literature. *Pediatr. Infect. Dis. J.* **2004***, 23*, 877-9.

231. Koren, G.; Polacheck, I.; Kaplan, H. Invasive mucormycosis in a non-immunocompromised patient. *J. Infect.* **1986**, 12, 165-7.
232. Kosmidis, C.; Katsogianni, K.; Matsoukas, S.; Tsagarakis, M.; Georgiou, P.; Giannopoulou P.; *et al*. A fatal case of cutaneous zygomycosis in a patient with severe metabolic acidosis. *Mycoses* **2009***, 52,* 364-7.
233. Kothari, A.; Shalin, S.C.; Crescencio, J.C.; Burgess, M.J. Skin lesion in a patient with acute myeloid leukemia. *Transpl. Infect. Dis.* **2016***, 19*.
234. Kramer, B.S.; Hernandez, A.D.; Reddick, R.L.; Levine, A.S. Cutaneous infarction. Manifestation of disseminated mucormycosis. *Arch. Dermatol.* **1977***, 113*, 1075-6.
235. Kraut, EJ.; Jordan, M.H.; Steiner, C.R. 3rd. Arterial occlusion and progressive gangrene caused by mucormycosis in a patient with burns. *J. Burn Care Rehabil.* **1993***, 14*, 552-6.
236. Kucinskiene, V.; Sutkute, A.; Valiukeviciene, S. Cutaneous fungal infection in a neonatal intensive care unit patient: a case report and literature review. *Pediatr. Dermatol.* **2014***, 31*, 267-70.
237. Kueht, M.; Villarreal, JA.; Reece, E.; Galvan, NTN.; Mysore, K.; Restrepo, A.; *et al*. Cutaneous mucormycosis in solid organ transplant recipients after hurricane Harvey: short- and long-term management. Plast. Reconstr. *Surg. Glob. Open.* **2019***, 7, e2041*.
238. Kumar, V.; Aggarwal, A.; Taneja, R.; Saha, S.S.; Khazanchi, R.K.; Kler, N.; Saluja, S. Primary cutaneous mucormycosis in a premature neonate and its management by tumescent skin grafting. *Br. J. Plast. Surg.* **2005***, 58*, 852-4.

239. Kumar, A.; Khilnani, G.C.; Aggarwal, S.; Kumar, S.; Banerjee, U.; Xess, I. Primary cutaneous mucormycosis in an immunocompetent host: report of a case. *Surg. Today* **2003***, 33*, 319-22.

240. Kumar, A.R.; Hunt, P.; Ritter, E.M.; Howard, R. Successful knee extensor mechanism reconstruction in a warfare-related open lower extremity injury complicated by mucormycosis infection: a case report. *J. Orthop. Trauma* **2012***, 26*, e7-10.

241. Kumbla, PA.; Lee, N.; Kimbrough, M.K. Mucormycosis of the forehead and sinuses in a trauma patient. *Plast. Reconstr. Surg. Glob. Open* **2016***, 4*, e818.

242. Kyriopoulos, E.J.; Kyriakopoulos, A.; Karonidis, A.; Gravvanis, A.; Gamatsi, I.; Tsironis C.; Tsoutsos, D. Burn injuries and soft tissue traumas complicated by mucormycosis infection: a report of six cases and review of the literature. *Ann. Burns Fire Disasters* **2015***, 28*, 280-7.

243. Lakshmi, V.; Rani, T.S.; Sharma, S.; Mohan, V.S.; Sundaram, C.; Rao, R.R.; Satyanarayana G. Zygomycotic necrotizing fasciitis caused by *Apophysomyces elegans*. *J. Clin. Microbiol.* **1993***, 31,* 1368-9.

244. Larché, J.; Machouart, M.; Burton, K.; Collomb, J.; Biava, MF.; Gérard, A.; Fortier, B. Diagnosis of cutaneous mucormycosis due to *Rhizopus microsporus* by an innovative PCR-restriction fragment-length polymorphism method. *Clin. Infect. Dis.* **2005***, 41,* 1362-5.

245. Laza-Stanca, V.; Reid, L.; Watson, J.D.; Williamson, E.C. Successful treatment of cutaneous zygomycosis with extensive surgical debridement and oral posaconazole in an immunocompetent patient. *J. Plast. Reconstr. Aesthet. Surg.* **2012***, 65*, 1259-61.

246. Lebeau, O.; Van Delden, C.; Garbino, J.; Robert, J.; Lamoth, F.; Passweg, J.; Chalandon, Y. Disseminated *Rhizopus microsporus* infection cured by salvage

allogeneic hematopoietic stem cell transplantation, antifungal combination therapy, and surgical resection. *Transpl. Infect. Dis.* **2010**, *12*, 269-72.

247. Lechevalier, P.; Hermoso, D.G.; Carol, A.; Bonacorsi, S.; Ferkdadji, L.; Fitoussi, F, *et al*. Molecular diagnosis of *Saksenaea vasiformis* cutaneous infection after scorpion sting in an immunocompetent adolescent. *J. Clin. Microbiol.* **2008**, *46*, 3169-72.

248. Ledgard, J.P.; van Hal, S.; Greenwood, J.E. Primary cutaneous zygomycosis in a burns patient: a review. *J. Burn Care Res.* **2008**, *29*, 286-90.

249. Leitner, C.; Hoffmann, J.; Zerfowski, M.; Reinert, S. Mucormycosis: necrotizing soft tissue lesion of the face. *J. Oral Maxillofac. Surg.* **2003**, *61*, 1354-8.

250. Lelievre, L.; Garcia-Hermoso, D.; Abdoul, H.; Hivelin, M.; Chouaki, T.; Toubas, D.; Mamez, A.C.; Lantieri, L.; Lortholary, O.; Lanternier, F; and the French Mycosis Study Group. Posttraumatic mucormycosis: a nationwide study in France and review of the literature. *Medicine* **2014**, *93*, 395-404.

251. LeMaile-Williams M.; Burwell L.A.; Salisbury D.; Noble-Wang J.; Arduino M.; Lott T, *et al.* Outbreak of cutaneous *Rhizopus arrhizus* infection associated with karaya ostomy bags. *Clin. Infect. Dis.* **2006**, *43,* e83-8.

252. Lenane, P.; Keane C.O.; Loughlin, S.O. Mucor mycosis infection presenting as a non-healing ulcer in an immunocompromised patient. *Clin. Exp. Dermatol.* **2003**, *28*, 157-9.

253. Leong, K.W.; Crowley, B.; White, B.; Crotty, G.M.; O'Briain, D.S.; Keane, C.; McCann, S.R. Cutaneous mucormycosis due to *Absidia corymbifera* occurring after bone marrow transplantation. *Bone Marrow Transplant.* **1997**, *19*, 513-5.

254. Lesueur, B.W.; Warschaw, K.; Fredrikson, L. Necrotizing cellulitis caused by *Apophysomyces elegans* at a patch test site. *Am. J. Contact Dermat.* **2002**, *13*, 140-2.

255. Lewandowski, L.; Purcell, R.; Fleming, M, Gordon, W.T. The use of dilute Dakin's solution for the treatment of angioinvasive fungal infection in the combat wounded: a case series. *Mil. Med.* **2013***, 178*, e503-7.

256. Li, F.; Yang, H.M.; Chai, J.K.; Wang, H.W. Burn wound mucormycosis: a case report. *J. Burn Care Res.* **2012***, 33*, e24-5.

257. Li, D.M.; Lun, LD. *Mucor irregularis* infection and lethal midline granuloma: a case report and review of published literature. *Mycopathologia* **2012***, 174*, 429-39.

258. Li, H.; Hwang, S.K.; Zhou, C.; Du, J.; Zhang, J. Gangrenous cutaneous mucormycosis caused by *Rhizopus oryzae*: a case report and review of primary cutaneous mucormycosis in China over Past 20 years. *Mycopathologia* **2013***, 176*, 123-8.

259. Liang, En W.; Seow, Yen T.; Ai Ling, T.; Yen Ee, T.; Sze Hwa, T.; Chun, A.C.; *et al*. Disseminated mucormycosis due to *Saksenaea vasiformis* complex in an immunocompetent adult with sustained response to posaconazole treatment. *Mycopathologia* **2020***, 185*, 577-81.

260. Liang, G.Z.; Xu, W.Q.; Zheng, X.L.; Mei, H.; Lv, G.X.; Shen, Y.N.; *et al*. Successful treatment by surgery of a primary cutaneous mucormycosis caused by *Mucor irregularis*. *Mycopathologia* **2018***, 183*, 445-9.

261. Lidor, C.; Nunley, J.A. Images in clinical medicine. Mucormycosis of the hand and forearm. *N. Engl. J. Med.* **1997***, 337*, 1511.

262. Linder, N.; Keller, N.; Huri, C.; Kuint, J.; Goldshmidt-Reuven, A.; Barzilai, A. Primary cutaneous mucormycosis in a premature infant: case report and review of the literature. *Am. J. Perinatol.* **1998***, 15*, 35-8.

263. Lineberry, K.D.; Boettcher, A.K.; Blount, A.L.; Burgess, S.D. Cutaneous mucormycosis of the upper extremity in an immunocompetent host: case report. *J. Hand Surg. Am.* **2012***, 37*, 787-91.

264. Liu, Z.H.; Lv, G.X.; Chen, J.; Sang, H.; She, X.D.; Chen, X.J.; *et al*. Primary cutaneous zygomycosis due to Absidia corymbifera in a patient with cutaneous T cell lymphoma. *Med. Mycol.* **2009***, 47*, 663-8.

265. Loganathan, S.; Ajay, G.A.E.; Thyagarajan, U.; Gokul, R.D. Invasive fungal infection in immunocompetent trauma patients - A case series. *J. Clin. Orthop. Trauma* **2018***, 9(Suppl 1)*, S10-S14.

266. London, V.; Chong, S. Cutaneous mucormycosis: a case report. Cutis 2012, 89, 167-8.

267. Lopes, J.O.; Pereira, D.V.; Streher, L.A.; Fenalte, A.A.; Alves, S.H.; Benevenga, J.P. Cutaneous zygomycosis caused by Absidia corymbifera in a leukemic patient. *Mycopathologia* **1995***, 130*, 89-92.

268. Losee, J.E.; Selber, J.; Vega, S.; Hall, C.; Scott, G.; Serletti, J.M. Primary cutaneous mucormycosis: guide to surgical management. *Ann. Plast. Surg.* **2002***, 49*, 385-90.

269. Lowe, C.D.; Sainato, R.J.; Stagliano, D.R.; Morgan, M.M.; Green, B.P. Primary cutaneous mucormycosis in an extremely preterm infant successfully treated with liposomal amphotericin B. *Pediatr. Dermatol.* **2017***, 34,* e116-9.

270. Lu, X.L.; Liu, Z.H.; Shen, Y.N.; She, X.D.; Lu, G.X.; Zhan, P.; *et al*. Primary cutaneous zygomycosis caused by *Rhizomucor variabilis*: a new endemic zygomycosis? A case report and review of 6 cases reported from China. *Clin. Infect. Dis* **2009***, 49*, e39-43.

271. Lu, D.; Hua, C.; Servy, A.; Foulet, F.; Botterel, F.; De Prost, N.; *et al*. Primary cutaneous mucormycosis as a complication of erosive dermatitis: two cases. *Eur. J. Dermatol.* **2018***, 28*, 227-9.

272. Lucas, J.B.; Salyer, R.D.; Watson, D.W. Gangrenous primary cutaneous mucormycosis of the scalp in an iatrogenically immunosuppressed trauma patient. *Otolaryngol. Head Neck Surg.* **2003***, 128*, 912-4.

273. Lumbang, W.A.; Caufield, B.A. Vesicular eruption on the arm of an infant. *Dermatol Online J.* **2010***, 16(5),*13.

274. Luo, Y.; Zeng, F.; Huang, X.; Li, Q.; Tan, G.; Xi, L, *et al*. Successful treatment of a necrotizing fasciitis patient caused by *Mucor indicus* with amphotericin B and skin grafting. *Mycopathologia* **2014***, 177*, 187-92.

275. Lye, G.R.; Wood, G.; Nimmo, G. Subcutaneous zygomycosis due to *Saksenaea vasiformis*: rapid isolate identification using a modified sporulation technique. *Pathology* **1996***, 28*, 364-5.

276. Maertens, J.; Demuynck, H.; Verbeken, E.K.; Zachée, P.; Verhoef, G.E.; Vandenberghe, P.; Boogaerts, M.A. Mucormycosis in allogeneic bone marrow transplant recipients: report of five cases and review of the role of iron overload in the pathogenesis. *Bone Marrow Transplant.* **1999***, 24*, 307-12.

277. Maffini, F.; Cocorocchio, E.; Pruneri, G.; Bonomo, G.; Peccatori, F.; Chiapparini, L.; *et al*. Locked-in syndrome after basilary artery thrombosis by mucormycosis masquerading as meningoencephalitis in a lymphoma patient. *Ecancermedicalscience* **2013***, 7*, 382.

278. Maleitzke, T.; Stahnke, K.; Trampuz, A.; Märdian, S. A case report of cutaneous mucormycosis of the hand after minor trauma in a patient with acute myeloid leukaemia. *Trauma Case Rep.* **2019***, 23*, 100221.

279. Mangaraj, S.; Sethy, G.; Patro, M.K.; Padhi, S. A rare case of subcutaneous mucormycosis due to *Syncephalastrum racemosum*: case report and review of literature. *Indian J. Med. Microbiol.* **2014***, 32*, 448-51.

280. Marchevsky, A.M.; Bottone, E.J.; Geller, S.A.; Giger, D.K. The changing spectrum of disease, etiology, and diagnosis of mucormycosis. *Hum. Pathol.* **1980***, 11*, 457-64.

281. Marcó del Pont, J.; De Cicco, L.; Gallo, G.; Llera, J.; De Santibanez, E.; D'agostino, D. Hepatic arterial thrombosis due to *Mucor* species in a child following orthotopic liver transplantation. *Transpl. Infect. Dis.* **2000***, 2,* 33-5.

282. Martín, L.B.; Rodríguez, M.Á.M.; Mercier, N.; Lafont, M.O.; Fernández, E.O.; de la Parte, A.R.; Estefanía, M. *Rhizopus arrhizus* invasive infection due to self-inflicted scratch injuries in a diabetic patient with non-ketotic acidosis. *Mycopathologia* **2017***, 182*, 927-31.

283. Martinez-Mugica, C.; Alba, S.R.; Boga, J.A.; Rodriguez-Guardado, A. Cutaneous infection due to *Mucor irregularis* (*Rhizomucor variabilis*) in an immunocompetent traveller. *Enferm. Infecc. Microbiol. Clin.* **2017***, 35,* 56-7.

284. Mata-Essayag, S.; Magaldi, S.; de Capriles, C.H.; Henao, L.; Garrido, L.; Pacillo, V. *Mucor indicus* necrotizing fasciitis. *Int. J. Dermatol.* **2001***, 40*, 406-8.

285. Mater, A.; Al-Sulaiti, G.; Johnston, D.L.; Slinger, R. A 4-year-old child with leukemia and an enlarging arm lesion. *C.M.A.J.* **2005***, 172*, 332.

286. Mathews, M.S.; Raman, A.; Nair, A. Nosocomial zygomycotic post-surgical necrotizing fasciitis in a healthy adult caused by *Apophysomyces elegans* in south India. *J. Med. Vet. Mycol.* **1997***, 35*, 61-3.

287. Matsudate, Y.; Murao, K.; Urano, Y.; Yarita, K.; Kamei, K.; Takeichi, H.; Kubo, Y. Primary cutaneous mucormycosis caused by *Mucor irregularis* in an immunocompetent patient. *J. Dermatol.* **2015***, 42*, 267-8.

288. McKenzie, S.; Zang, P.; Blackcloud, P.; Cohen, B.; Truong, A.; Worswick, S.; Arzeno, J. Case series of cutaneous mucormycosis in the setting of Herpesviridae infection. *Br. J. Dermatol.* **2019***, 181*, 373-4.

289. McSpadden, R.P.; Martin, J.R.; Mehrotra, S.; Thorpe, E. Mucormycosis causing Ludwig angina: a unique presentation*. J. Oral Maxillofac. Surg.* **2016***, 75*, 759-62.

290. Mead, J.H.; Lupton, G.P.; Dillavou, C.L.; Odom, R.B. Cutaneous *Rhizopus* infection. Occurrence as a postoperative complication associated with an elasticized adhesive dressing. *J.A.M.A* **1979***, 242*, 272-4.

291. Melsom, S.M.; Khangure, M.S. Craniofacial mucormycosis following assault: an unusual presentation of an unusual disease. *Australas. Radiol.* **2000***, 44*, 104-6.

292. Menzinger, S.; Sid'Amar, S.; Kaya, G. Cutaneous mucormycosis resulting from hematogenous dissemination of *Rhizomucor pusillus* in an immunocompromised patient. *Dermatopathology* **2020***, 6,* 275-78.

293. Meyer, R.D.; Kaplan, M.H.; Ong, M.; Armstrong, D. Cutaneous lesions in disseminated mucormycosis. *J.A.M.A* **1973***, 225*, 737-8.

294. Mishra, S.; Shelly, D.; Gupta, D.; Bharadwaj, R. Invasive cutaneous mucormycosis in a preterm neonate presenting as a vesicobullous lesion. *Indian J. Pathol. Microbiol.* **2018,** 61:103-5.

295. Mitchell, S.J.; Gray, J.; Morgan, M.E.; Hocking, M.D.; Durbin, G.M. Nosocomial infection with *Rhizopus microsporus* in preterm infants: association with wooden tongue depressors. *Lancet* **1996***, 348*, 441-3.

296. Miyamoto, H.; Hayashi, H.; Nakajima, H. Cutaneous mucormycosis in a patient with acute lymphocytic leukemia. *J. Dermatol.* **2005***, 32*, 273-7.

297. Mizutari, K.; Nishimoto, K.; Ono, T. Cutaneous mucormycosis. *J. Dermatol.* **1999***, 26*, 174-7.

298. Moon, P.; Jithendran, N. Invasive Fungal Infection with *Absidia Corymbifera* in Immunocompetent Patient with Electrical Scalp Burn. *World J. Plast. Surg.* **2018***, 7*, 249-52.

299. Morado-Aramburo, O.; Ortiz-Brizuela, E.; Méndez-Flores, S.; Cuellar-Rodríguez, J. Necrotic skin ulcers in an immunocompromised patient. *Enferm. Infecc. Microbiol. Clin. (Engl Ed).* **2019***, 37,* 476-9.

300. Morales-Aguirre, J.J.; Agüero-Echeverría, W.M.; Ornelas-Carsolio, M.E.; Reséndiz-Sánchez, J.; Gómez-Barreto, D.; Cashat-Cruz, M. Successful treatment of a primary cutaneous zygomycosis caused by Absidia corymbifera in a premature newborn. *Pediatr. Infect. Dis. J.* **2004***, 23*, 470-2.

301. Moran, S.L.; Strickland, J.; Shin, A.Y. Upper-extremity mucormycosis infections in immunocompetent patients. *J. Hand Surg. Am.* **2006***, 31*, 1201-5.

302. Moreira J.; Ridolfi F.; Almeida-Paes R.; Varon A.; Lamas C.C. Cutaneous mucormycosis in advanced HIV disease. *Braz. J. Infect. Dis.* **2016***, 20*, 637-40.

303. Mostaza, J.M.; Barbado, F.J.; Fernandez-Martin, J.; Peña-Yañez, J.; Vazquez-Rodriguez, J.J. Cutaneoarticular mucormycosis due to *Cunninghamella bertholletiae* in a patient with AIDS. *Rev. Infect. Dis.* **1989***, 11,* 316-8.

304. Motohashi, K.; Ito, S.; Hagihara, M.; Maruta, A.; Ishigatsubo, Y.; Kanamori, H. Cutaneous zygomycosis caused by *Cunninghamella bertholletiae* in a patient with chronic myelogenous leukemia in blast crisis. *Am. J. Hematol.* **2009***, 84,* 447-8.

305. Mueller, B.U.; Pabst, T. Cutaneous zygomycosis at catheter insertion site in AML-M4Eo. *Ann. Hematol.* **2006***, 85*, 194-5.

306. Mukherjee, B.; Kundu, D. Necrotizing fungal infection due to *Saksenaea erythrospora*: a case report and review of the literature. *Indian J. Ophthalmol.* **2018**, *66*, 1513-6.

307. Myskowski, P.L.; Brown, A.E.; Dinsmore, R.; Kiehn, T.; Edwards, F.; Wong, B.; *et al*. Mucormycosis following bone marrow transplantation. *J. Am. Acad. Dermatol.* **1983***, 9,* 111-5.

308. Naguib, M.T.; Huycke, M.M.; Pederson, J.A.; Pennington, L.R.; Burton, M.E.; Greenfield, RA. *Apophysomyces elegans* infection in a renal transplant recipient. *Am. J. Kidney Dis.* **1995***, 26*, 381- 4.

309. Nain, P.S.; Matta, H.; Singh, K.; Chhina, D.; Trehan, M.; Batta, N. Post-operative abdominal wall mucormycosis-a case series. *Indian J. Surg.* **2015***, 77(Suppl 2),* 253-6.

310. Narayanan, M.l. S.; Narayanan, C.D.; Kindo, A.J.; Arora, A.; Haridas, P.A. Fatal fungal infection: the living dead. *J. Surg. Case Rep.* **2014***, 2014*, rju104.

311. Neblett Fanfair, R.; Benedict, K.; Bos, J.; Bennett, S.D.; Lo, Y.C.; Adebanjo, T.; *et al*. Necrotizing cutaneous mucormycosis after a tornado in Joplin, Missouri, in 2011. N *Engl. J. Med.* **2012***, 367*, 2214-25.

312. Newton, W.D.; Cramer, F.S.; Norwood, S.H. Necrotizing fasciitis from invasive Phycomycetes. *Crit. Care Med.* **1987***, 15*, 331-2.

313. Ng, P.C.; Dear, P.R. Phycomycotic abscesses in a preterm infant. *Arch. Dis. Child* **1989***, 64,* 862-4.

314. Nitinawarat, J.; Putcharoen, O.; Chindamporn, A.; Rerknimitr P. Subcutaneous *Saksenaea vasiformis* infection presenting as disfiguring facial plaques. *Indian J. Dermatol. Venereol. Leprol.* **2017**, *83*, 346-8.

315. Nordén, G.; Björck, S.; Persson, H.; Svalander, C.; Li, X.G.; Edebo, L. Cure of zygomycosis caused by a lipase-producing *Rhizopus rhizopodiformis* strain in a renal transplant patient. *Scand. J. Infect. Dis.* **1991**, *23*, 377-82.

316. Nouri-Majalan, N.; Moghimi, M. Skin mucormycosis presenting as an erythema-nodosum-like rash in a renal transplant recipient: a case report. *J. Med. Case Rep.* **2008**, *2*, 112.

317. Numa, W.A Jr.; Foster, P.K.; Wachholz, J.; Civantos, F.; Gomez-Fernandez, C.; Weed, D.T. Cutaneous mucormycosis of the head and neck with parotid gland involvement: first report of a case. *Ear Nose Throat J.* **2004**, *83*, 282, 284, 286.

318. Oberle, A.D.; Penn, R.L. Nosocomial invasive *Saksenaea vasiformis* infection. *Am. J. Clin. Pathol.* **1983**, *80*, 885-8.

319. Oh, D.; Notrica, D. Primary cutaneous mucormycosis in infants and neonates: case report and review of the literature. *J. Pediatr. Surg.* **2002**, *37*, 1607-11.

320. Okhuysen, P.C.; Rex, J.H.; Kapusta, M.; Fife, C. Successful treatment of extensive posttraumatic soft-tissue and renal infections due to *Apophysomyces elegans*. *Clin. Infect. Dis.* **1994**, *19*, 329-31.

321. De Oliveira-Neto, M.P.; Da Silva, M.; Fialho Monteiro, P.C.; Lazera, M.; de Almeida Paes, R.; Novellino, A.B.; Cuzzi, T. Cutaneous mucormycosis in a young, immunocompetent girl. *Med. Mycol.* **2006**, *44*, 567-70.

322. Padhye, A.A.; Koshi, G.; Anandi, V.; Ponniah, J.; Sitaram, V.; Jacob, M.; *et al*. First case of subcutaneous zygomycosis caused by *Saksenaea vasiformis* in India. *Diagn. Microbiol. Infect. Dis*. **1988***, 9*, 69-77.

323. Paduraru, M.; Moreno-Sanz, C.; Olalla Gallardo, J.M. Primary cutaneous mucormycosis in an immunocompetent patient. *B.M.J. Case Rep.* **2016***, 2016*, bcr2016214982.

324. Page, R.; Gardam, D.J.; Heath, C.H. Severe cutaneous mucormycosis (Zygomycosis) due to *Apophysomyces elegans*. *A.N.Z. J. Surg.* **2001***, 71*, 184-6.

325. Page, A.V.; Evans, A.J.; Snell, L.; Liles, W.C. Primary cutaneous mucormycosis in a lung transplant recipient: case report and concise review of the literature. *Transpl. Infect. Dis.* **2008***, 10*, 419-25.

326. Palmer, D.L.; Weitzner, S.; Simpson, J.C. Progressive gangrene of an extremity due to mucormycosis in a diabetic patient. *Diabetes* **1970***, 19*, 881-3.

327. Pamidimukkala, U.; Sudhaharan, S.; Kancharla, A.; Vemu, L.; Challa, S.; Karanam, S.D.; *et al*. Mucormycosis due to *Apophysomyces* species complex- 25 years' experience at a tertiary care hospital in southern India. *Med. Mycol.* **2020***, 58*, 425-33.

328. Paolino, K.M.; Henry, J.A.; Hospenthal, D.R.; Wortmann, G.W.; Hartzell J.D. Invasive fungal infections following combat-related injury. *Mil. Med.* **2012***, 177*, 681-5.

329. Paparello, S.F.; Parry, R.L.; MacGillivray, D.C.; Brock, N.; Mayers, D.L. Hospital-acquired wound mucormycosis. *Clin. Infect. Dis.* **1992***, 14*, 350-2.

330. Papastavros, V.; Nathoo, R.; Potter, K.A.; Gonzalez Santiago, T. A refractory ulcer in an immunocompromised patient: what caused it? *Int. J. Dermatol.* **2016***, 56*, 597-8.

331. Parker C.; Kaminski G.; Hill D. Zygomycosis in a tattoo, caused by *Saksenaea vasiformis*. *Australas. J. Dermatol.* **1986***, 27*, 107-11.

332. Parkyn, T.; McNinch, A.W.; Riordan, T.; Mott, M. Zygomycosis in relapsed acute leukaemia. *J. Infect.* **2000***, 41*, 265-8.

333. Pasticci, M.B.; Terenzi, A.; Lapalorcia, L.M.; Giovenale, P.; Pitzurra, L.; Constantini, V.; Lignani, A.; Gurdo, G.; Verzini, F.; Baldelli, F. *Absidia corymbifera* necrotizing cellulitis in an immunocompromised patient while on voriconazole treatment. *Ann. Hematol*. **2008**, *87,* 687-9.

334. Patil, A.B.; Chandramohan, K.; Shivaprakash, M.R.; Nadgir, S.D.; Lakshminarayana, S.A. *Rhizomucor variabilis*: a rare causative agent of primary cutaneous zygomycosis. *Indian J. Med. Microbiol.* **2013***, 31*, 302-5.

335. Patiño, J.F.; Mora, R.; Guzmán, M.A.; Rodríguez-Franco, E. Mucormycosis: a fatal case by *Saksenaea vasiformis*. *World J. Surg.* **1984***, 8*, 419-22.

336. Patterson, J.E.; Barden, G.E.; Bia, FJ. Hospital-acquired gangrenous mucormycosis. *Yale J. Biol. Med* **1986***, 59*:453-9.

337. Peñas, P.F.; Rios, L.; de la Cámara, R.; Fraga, J.; Daudén, E. Cutaneous lesions as the first sign of disseminated mucormycosis. *Acta Derm. Venereol.* **1995***, 75*, 166-7.

338. Pepeler, M.S.; Acar, K.; Güzel Tunçcan, Ö.; Uluoğlu, Ö.; Kalkancı, A.; Atalar, H.; *et al*. A proven case of cutaneous *Rhizopus* infection presenting with severe limb pain very soon after induction treatment in a patient with acute lymphoblastic leukemia. *Case Rep. Hematol.* **2015***, 2015,* 285360.

339. Perz, A.; Makar, G.; Fernandez, E.; Weinstock, J.; Rafferty ,W. Primary cutaneous mucormycosis of the abdomen at the site of repeated insulin injections. *B.M.J. Case Rep.* **2020***, 13*, e233284.

340. Piazza, R.C.; Thomas, W.L.; Stawski, W.S.; Ford, R.D. Mucormycosis of the face. *J. Burn Care Res.* **2009***, 30*, 520-3.

341. Pierce, P.F.; Wood, M.B.; Roberts, G.D.; Fitzgerald, R.H Jr.; Robertson, C.; Edson, R.S. *Saksenaea vasiformis* osteomyelitis. *J. Clin. Microbiol.* **1987***, 25*, 933-5.

342. Pilch, W.T.; Kinnear, N.; Hennessey, D.B. *Saksenaea vasiformis* infection in an immunocompetent patient in rural Australia. *B.M.J. Case Rep.* **2017***, 2017,* bcr2017220341.

343. Poirier, P.; Nourrisson, C.; Gibold, L.; Chalus, E.; Guelon, D.; Descamp, S.; *et al*. Three cases of cutaneous mucormycosis with *Lichtheimia* spp. (ex *Absidia/Mycocladus*) in ICU. Possible cross-transmission in an intensive care unit between 2 cases. *J. Mycol. Med.* **2013***, 23,* 265-9.

344. Pourahmad, M.; Sepidkar, A.; Farokhnia, M.H.; Tadayon, S.M.; Salehi, H.; Zabetian, H. Mucormycosis after scorpion sting: case report. *Mycoses* **2013***, 56*, 589-91.

345. Prevoo, R.L.; Starink, T.M.; de Haan, P. Primary cutaneous mucormycosis in a healthy young girl. Report of a case caused by *Mucor hiemalis Wehmer*. *J. Am. Acad. Dermatol.* **1991***, 24*, 882-5.

346. Pritchard, R.C.; Muir, D.B.; Archer, K.H.; Beith, J.M. Subcutaneous zygomycosis due to *Saksenaea vasiformis* in an infant. *Med. J. Aust.* **1986***, 145*, 630-1.

347. Prokopowicz, G.P.; Bradley, S.F.; Kauffman, C.A. Indolent zygomycosis associated with deferoxamine chelation therapy. *Mycoses* **1994***, 37*, 427-31.

348. Quinio, D.; Karam, A.; Leroy, J.P.; Moal, M.C.; Bourbigot, B.; Masure, O.; *et al*. Zygomycosis caused by *Cunninghamella bertholletiae* in a kidney transplant recipient. *Med. Mycol.* **2004***, 42*, 177-80.

349. Radowsky, J.S.; Strawn, A.A.; Sherwood, J.; Braden, A.; Liston, W. Invasive mucormycosis and aspergillosis in a healthy 22-year-old battle casualty: case report. *Surg. Infect.* **2011***, 12*, 397-400.

350. Raizman, N.M.; Parisien, M.; Grafe, M.W.; Gordon, R.J.; Rosenwasser, M.P. Mucormycosis of the upper extremity in a patient with alcoholic encephalopathy. *J. Hand Surg. Am.* **2007**, *32,* 384-8.

351. Rammaert, B.; Angebault, C.; Scemla, A.; Fraitag, S.; Lerolle, N.; Lecuit, M.; *et al.* *Mucor irregularis*-associated cutaneous mucormycosis: Case report and review. *Med. Mycol. Case Rep.* **2014**, *6*, 62-5.

352. Rangel-Guerra, R.; Martínez, HR.; Sáenz, C. Mucormycosis. Report of 11 cases. *Arch. Neurol.* **1985**, *42*, 578-81.

353. Razmi, T.M.; Shivaprakash, M.R.; Saikia, U.N.; De, D.; Handa, S. "All That Necroses Is Not Toxic Epidermal Necrolysis". *J. Cutan. Med. Surg.* **2017**, *21*, 172-3.

354. Reddy, I.S.; Rao, N.R.; Shankar Reddy, V.M.; Rao R. Primary cutaneous mucormycosis (zygomycosis) caused by *Apophysomyces elegans*. *Indian J. Dermatol. Venereol. Leprol.* **2008**, *74*, 367-70.

355. Reich, P.; Shute, T.; Lysen, C.; Lockhart, SR.; Keating M.K; Custer P.; Orscheln R. *Saksenaea vasiformis* orbital cellulitis in an immunocompetent child treated with posaconazole. *J. Pediatric Infect. Dis. Soc.* **2018**, *7*, e169-71.

356. Reinbold, C.; Derder, M.; Hivelin, M.; Ozil, C.; Al Hindi, A.; Lantieri, L. Using free flaps for reconstruction during infections by mucormycosis: A case report and a structured review of the literature. *Ann. Chir. Plast. Esthet.* **2016**, *61*, 153-61.

357. Relloso, S.; Romano, V.; Landaburu, M.F.; Herrera, F.; Smayevsky, J.; Veciño, C.; Mujica, MT. *Saksenaea erythrospora* infection following a serious sailing accident. *J. Med. Microbiol.* **2014**, *63*, 317-21.

358. Requena, L.; Sitthinamsuwan, P.; Santonja, C.; Fernández-Figueras, M.T.; Rodríguez-Peralto, J.L.; *et al*. Cutaneous and mucosal mucormycosis mimicking pancreatic panniculitis and gouty panniculitis. J Am Acad Dermatol 2012, 66, 975-84.

359. Ribeiro, N.F.; Cousin, G.C.; Wilson, G.E.; Butterworth, D.M.; Woodwards, R.T. Lethal invasive mucormycosis: case report and recommendations for treatment. *Int. J. Oral Maxillofac. Surg.* **2001**, *30*, 156-9.

360. Righi, E.; Giacomazzi, C.G.; Bassetti, M.; Bisio, F.; Soro, O.; McDermott, J.L.; *et al*. Soft-tissue infection with *Absidia corymbifera* and kidney complications in an AIDS patient. *Med. Mycol.* **2007**, *45*, 637-40.

361. Roberts, H.J. Cutaneous mucormycosis. Report of a case with survival. *Arch. Intern. Med.* **1962**, *110*, 108-12.

362. Rodríguez, J.Y.; Rodríguez, G.J.; Morales-López, S.E.; Cantillo, C.E.; Le Pape, P.; Álvarez-Moreno, C.A. *Saksenaea erythrospora* infection after medical tourism for esthetic breast augmentation surgery. *Int. J. Infect. Dis.* **2016**, *49*, 107-10.

363. Rodríguez-Lobato, E.; Ramírez-Hobak, L.; Aquino-Matus, J.E.; Ramírez-Hinojosa, J.P.; Lozano-Fernández, V.H.; Xicohtencatl-Cortes, J.; *et al*. Primary cutaneous mucormycosis caused by *Rhizopus oryzae*: a case report and review of literature. *Mycopathologia* **2017**, *182*, 387-92.

364. Rodríguez, J.Y.; Morales-López, S.E.; Rodríguez, G.J.; Álvarez-Moreno, C.A.; Ocampo, W.; Cepeda, M.L.; Mora-Valderrama, M.A. Necrotizing fasciitis caused by *Apophysomyces variabilis* in an immunocompetent patient. *Med. Mycol. Case Rep.* **2017**, *20*, 4-6.

365. Romano, C.; Ghilardi, A.; Massai, L.; Capecchi, P.L.; Miraccco, C.; Fimiani, M. Primary subcutaneous zygomycosis due to *Rhizopus oryzae* in a 71-year-old man with normal immune status. *Mycoses* **2007***, 50*, 82-4.

366. Rothburn, M.M.; Chambers, D.K.; Roberts, C.; Downie, R.J. Cutaneous mucormycosis: a rare cause of leg ulceration. J. *Infect.* **1986***, 13*, 175-8.

367. Roux, B.G.; Méchinaud, F.; Gay-Andrieu, F.; Lortholary, O.; Dannaoui, E.; Hoinard, D.; Corradini, N. Successful triple combination therapy of disseminated absidia corymbifera infection in an adolescent with osteosarcoma. *J. Pediatr. Hematol. Oncol.* **2010***, 32,* 131-3.

368. Rubin, AI.; Grossman, M.E. Bull's-eye cutaneous infarct of zygomycosis: a bedside diagnosis confirmed by touch preparation. *J. Am. Acad. Dermatol.* **2004***, 51*, 996-1001.

369. Ryan-Poirier, K.; Eiseman, R.M.; Beaty, J.H.; Hunt, P.G.; Burghen, G.A.; Leggiadro, R.J. Post-traumatic cutaneous mucormycosis in diabetes mellitus. Short-term antifungal therapy. *Clin. Pediatr.* **1988***, 27*, 609-12.

370. Ryan, ME.; Ochs, D.; Ochs, J. Primary cutaneous mucormycosis: superficial and gangrenous infections. *Pediatr. Infect. Dis.* **1982***, 1*, 110-4.

371. Sagar, S.; Kumar, S.; Gupta, A.; Singhal, M.; Mishra, B. Successful management of a case of primary cutaneous mucormycosis in an immunocompetent host. *Wounds* **2013***, 25,* 136-40.

372. Sağıroğlu, P.; Nedret, Koç, A.; Atalay, M.A.; Altinkanat, Gelmez, G.; Canöz, Ö, Mutlu Sarıgüzel, F. Mucormycosis experience through the eyes of the laboratory. *Infect. Dis.* **2019***, 51*, 730-7.

373. Sahuquillo-Torralba, A.; Garrido-Jareño, M.; Llavador-Ros, M.; Botella-Estrada, R. Rapidly progressive frontal necrotic plaque in an immunosuppressed host. *Enferm. Infecc. Microbiol. Clin.* **2018**, *36*, 315-6.

374. Sahuquillo-Torralba, A.; Calle-Andrino, A.; Navarro-Mira, M.Á.; Llavador-Ros, M.; Botella-Estrada, R. Acute necrotic plaque in an immunocompromised host. *Am. J. Dermatopathol.* **2017**, *39*, 369.

375. Salisbury, R.E.; Silverstein, P.; Goodwin, M.N Jr. Upper extremity fungal invasions secondary to large burns. *Plast. Reconstr. Surg.* **1974**, *54*, 654-9.

376. Samaddar, A.; Sharma, A.; Maurya, V.K.; Tak, V. Necrotizing fasciitis caused by *Apophysomyces variabilis* in a burn patient. *I.D. Cases* **2019**, *18*, e00660.

377. Samaras, K.; Markantonatou, A.M.; Karapiperis, D.; Digonis, P.; Kartalis, N.; Kostogloudis, N.; Vyzantiadis, T.A. *Saksenaea vasiformis* infections: A case of an immunocompetent adult after mild injury and a literature review. *J. Mycol. Med.* **2019**, *29*, 260-4.

378. Sanchez, M.R.; Ponge-Wilson, I.; Moy, J.A.; Rosenthal, S. Zygomycosis and HIV infection. *J. Am. Acad. Dermatol.* **1994**, *30*, 904-8.

379. Sankar, J.; Arun, S.; Sankar, M.J.; Seth, R.; Thavraj, V.; Kabra, S.K.; Vasantha, M. Primary cutaneous mucormycosis during induction chemotherapy in a child with acute lymphoblastic leukemia. *Indian J. Pediatr.* **2009**, *76*, 1161-3.

380. Saravia-Flores, M.; Guaran, D.M.; Argueta, V. Invasive cutaneous infection caused by *Apophysomyces elegans* associated with a spider bite. *Mycoses* **2010**, *53*, 259-61.

381. Sawardekar, K.P. Gangrenous necrotizing cutaneous mucormycosis in an immunocompetent neonate: A Case Report from Oman. *J. Trop. Pediatr.* **2018**, *64*, 548-52.

382. Scalise, A.; Barchiesi, F.; Viviani, M.A.; Arzeni, D.; Bertani, A.; Scalise, G. Infection due to *Absidia corymbifera* in a patient with a massive crush trauma of the foot. *J. Infect.* **1999***, 38*, 191-2.

383. Scheffler, E.; Miller, G.G.; Classen, D.A. Zygomycotic infection of the neonatal upper extremity. *J. Pediatr. Surg.* **2003***, 38*, E16-7.

384. Schell, W.A.; O'Donnell, K.; Alspaugh, J.A. Heterothallic mating in *Mucor irregularis* and first isolate of the species outside of Asia. *Med. Mycol.* **2011***, 49*, 714-23.

385. Schofield, C.; Stern, A.; Jevtic, A. Disseminated zygomycosis due to *Mycocladus corymbifera* with cutaneous and cerebral involvement. *Australas. J. Dermatol.* **2013***, 54,* e8-11.

386. Seguin, P.; Musellec, H.; Le Gall, F.; Chevrier, S.; Le Bouquin, V.; Malledant, Y. Post-traumatic course complicated by cutaneous infection with *Absidia corymbifera*. *Eur. J. Clin. Microbiol. Infect. Dis.* **1999***, 18*, 737-9.

387. Sękowska, A.; Prażyńska, M.; Twarużek, M.; Deptuła, A.; Zastempowska, E.; Soszczyńska, E.; Gospodarek-Komkowska, E. Fulminant mucormycosis after a traffic accident: a case report. *Folia Microbiol.* **2019***, 64*, 429-33.

388. Sepaskhah, M.; Moezzi, I.; Davarpanah, M.A.; Sari Aslani, F. Primary cutaneous mucormycosis in a beta-thalassemia patient. *Indian J. Hematol. Blood Transfus.* **2018***, 34*, 776-7.

389. Shah, A.; Lagvankar, S.; Shah, A. Cutaneous mucormycosis in children. *Indian Pediatr.* **2006***, 43,* 167-70.

390. Shakoor, S.; Jabeen, K.; Idrees, R.; Jamil, B.; Irfan, S.; Zafar, A. Necrotising fasciitis due to *Absidia corymbifera* in wounds dressed with non sterile bandages. *Int. Wound J.* **2011***, 8*, 651-5.

391. Sheldon, D.L.; Johnson, W.C. Cutaneous mucormycosis. Two documented cases of suspected nosocomial cause. *J.A.M.A.* **1979***, 241*, 1032-4.

392. Shiraishi, K.; Sasaki, S.; Sadamoto, Y. Cutaneous mucormycosis in a patient with acute lymphocytic leukemia. *Eur. J. Dermatol.* **2014***, 24*, 116-7.

393. Shivananda, P.; Mahabala, C.; Kausalya, S.; Suchitra, S.; Anand, K.U. Cutaneous mucormycosis with necrotising fasciitis in a young immunocompetent individual. *Trop. Doct.* **2011***, 41*, 183-4.

394. Sigera, L.S.M.; Gamage, K.K.K.; Jayawardena, M.N.; Abeydeera, W.P.H.; Malkanthi, M.A.; Jayasekera, P.I.; *et al*. Cutaneous mucormycosis caused by *Saksenaea vasiformis* in a patient with systemic lupus erythematosus. *Clin. Case Rep.* **2018***, 6*, 1730-4.

395. Simbli, M.; Hakim, F.; Koudieh, M.; Tleyjeh, I.M. Nosocomial post-traumatic cutaneous mucormycosis: a systematic review. *Scand. J. Infect. Dis.* **2008***, 40*, 577-82.

396. Singla, K.; Samra, T.; Bhatia, N. Primary cutaneous mucormycosis in a trauma patient with Morel-Lavallée lesion. *Indian J. Crit. Care Med.* **2018***, 22*, 375-7.

397. Sirignano, S.; Blake, P.; Turrentine, J.E.; Dominguez, A.R. Primary cutaneous zygomycosis secondary to minor trauma in an immunocompromised pediatric patient: a case report. *Dermatol. Online J.* **2014***, 20*, 13030/qt4r2455h9.

398. Sittig, K.R.; Laageide, L.G.; Akhtar, Z.; Wall, G.C.; Kumar, S.C. Cutaneous mucormycosis in a chronic lymphocytic leukemia patient on ibrutinib. *I.D. Cases* **2021***, 24*, e01120.

399. Snell, B.J.; Tavakoli, K. Necrotizing fasciitis caused by *Apophysomyces elegans* complicating soft-tissue and pelvic injuries in a tsunami survivor from Thailand. *Plast. Reconstr. Surg.* **2007***, 119*, 448-9.

Articles included in the analysis for the systematic review

400. Solano, T.; Atkins, B.; Tambosis, E.; Mann, S.; Gottlieb, T. Disseminated mucormycosis due to *Saksenaea vasiformis* in an immunocompetent adult. *Clin. Infect. Dis.* **2000**, *30*, 942-3.

401. Song, W.K.; Park, H.J.; Cinn, Y.W.; Rheem, I.; Pai, H.; Shin, J.H. Primary cutaneous mucormycosis in a trauma patient. *J. Dermatol.* **1999**, *26*, 825-8.

402. Song, K.R.; Wong, M.S.; Yeung, C. Primary cutaneous zygomycosis in an immunodeficient infant: a case report and review of the literature. *Ann. Plast. Surg.* **2008**, *60,* 433-6.

403. Srivastava, A.; Vij, V.; Wadhawan, M.; Gupta, S. Successful treatment of cutaneous mucormycosis infection following living donor liver transplant and review of literature. *Apollo Medicine* **2007**, *4*, 345-9.

404. Stanistreet, B.; Bell, D. Burn Wound Mucormycosis: A case study on poor wound healing. *J. Burn. Care Res.* **2017**, *38*, e582-4.

405. Stasiak, M.; Samet, A.; Lasek, J.; Wujtewicz, M.; Witkowski, Z.; Komarnicka, J.; *et al*. Mucormycosis complicating lower limb crash injury in a multiple traumatized patient: an unusual case. *B.M.J. Case Rep*. **2009,** 2009:bcr10.2008.1170.

406. Stein, M.K.; Karri, S.; Reynolds, J.; Owsley, J.; Wise, A.; Martin, MG.; Zare, F. Cutaneous mucormycosis following a bullous pemphigoid flare in a chronic lymphocytic leukemia patient on ibrutinib. *World J. Oncol.* **2018**, *9*, 62-5.

407. Stewardson, A.J.; Holmes, N.E.; Ellis, D.H.; Howden, B.P. Cutaneous zygomycosis caused by *Saksenaea vasiformis* following water-related wound in a 24-year-old immunocompetent woman. *Mycoses* **2009**, *52*, 547-9.

408. Strasfeld, L.; Espinosa-Aguilar, L.; Gajewski, J.L.; Stenzel, P.; Pimentel, A.; Mater, E.; Maziarz, R.T. Emergence of *Cunninghamella* as a pathogenic invasive mold infection in allogeneic transplant recipients. *Clin. Lymphoma Myeloma Leuk.* **2013***, 13*, 622-8.

409. Struck, M.F.; Illert, T.; Stiller, D.; Steen, M. Basilar artery occlusion after multifactor coagulopathy including Rhizopus oryzae infection in burns. *J. Burn Care Res.* **2010***, 31*, 955-8.

410. Sugui. J.A.; Christensen, J.A.; Bennett, J.E.; Zelazny, A.M.; Kwon-Chung, K.J. Hematogenously disseminated skin disease caused by Mucor velutinosus in a patient with acute myeloid leukemia. *J. Clin. Microbiol.* **2011***, 49*, 2728-32.

411. Sundararajan, T.; Kumar, CP.; Menon, T.; Rekha, K.; Venkatadesikalu, M. Cutaneous zygomycosis due to Rhizopus oryzae in a patient with acute lymphoblastic leukemia. *Mycoses* **2004***, 47*, 521-3.

412. Suo, L.; Dunn, J.J. The brief case: cutaneous fungal infection in a pediatric patient with newly diagnosed acute lymphocytic leukemia. J*. Clin. Microbiol.* **2020***, 58*, e00787-19.

413. Suthananthan, A.E.; Koek, S.A.; Sieunarine, K. Cutaneous mucormycosis in an immunocompromised patient: a case report. *J. Surg. Case Rep.* **2017***, 2017*, rjx056.

414. Takabayashi, M.; Sakai, R.; Sakamoto, H.; Kakinuma, M.; Iemoto, Y.; Kanamori, H.; Ishigatsubo, Y. Cutaneous mucormycosis during induction chemotherapy for acute lymphocytic leukemia. *Leuk. Lymphoma* **2004***, 45*, 199-200.

415. Tanabodee, M.; Sawasdipong, J.; Sudtikoonaseth, P.; Wessagowit, V. Rare indolent zygomycosis caused by subcutaneous *Saksenaea vasiformis* infection. *Australas. J. Dermatol.* **2020***, 61*, e94-6.

416. Tang, D.; Wang, W. Successful cure of an extensive burn injury complicated with mucor wound sepsis. *Burns* **1998***, 24*, 72-3.

417. Tang, X., Guo, P., Wong, H., Xie, J., Han, J., Xu, Y., & Zhou, H. Vacuum-assisted closure and skin grafting combined with amphotericin B for successful treatment of an immunocompromised patient with cutaneous mucormycosis caused by *Mucor irregularis*: a case report and literature review. *Mycopathologia,* **2021***, 186(3),* 449–59.

418. Tanphaichitr, V.S.; Chaiprasert, A.; Suvatte, V.; Thasnakorn, P. Subcutaneous mucormycosis caused by Saksenaea vasiformis in a thalassaemic child: first case report in Thailand. *Mycoses* **1990***, 33*, 303-9.

419. Tapish, S.; Taha, M.; Naresh, G.; Neeraj, D.; Malik Vinod, K. Primary mucormycosis of abdominal wall: A rare fungal infection in a immunocompetent patient. *Indian J. Surg.* **2010***, 72(Suppl 1),* 306-8.

420. Tehmeena, W.; Hussain, W.; Zargar, HR.; Sheikh, AR.; Iqbal, S. Primary cutaneous mucormycosis in an immunocompetent host. *Mycopathologia* **2007***, 164*, 197-9.

421. Tendolkar, U.; Baradkar, V.; Baveja, S.; Gore, M.; Bankar, S.; Gore, S. Cutaneous Zygomycosis due to *Saksenaea vasiformis* in a patient with paraparesis, burns and pressure ulcer. *Int. J. Infect. Dis.* **2012***, 10*, 1-4.

422. Thami, G.P.; Kaur, S.; Bawa, A.S.; Chander, J.; Mohan, H.; Bedi, M.S. Post-surgical zygomycotic necrotizing subcutaneous infection caused by *Absidia corymbifera*. *Clin. Exp. Dermatol.* **2003***, 28,* 251-3.

423. Thawani, R.; Singh, S.; Sharma, S.; Sharma, N. Infective gangrene in extremity trauma-are we targeting the right organisms? *Indian J. Surg.* **2015***, 77(Suppl 1),* 10-2.

424. Thielen, B.K.; Barnes, A.M.T.; Sabin, A.P.; Huebner, B.; Nelson, S.; Wesenberg, E.; Hansen, G.T. Widespread *Lichtheimia* Infection in a patient with extensive burns: opportunities for novel antifungal agents. *Mycopathologia* **2019***, 184*, 121-8.

425. Tidwell, J.; Higuera, S.; Hollier, L.H Jr. Facial reconstruction after mucormycosis in an immunocompetent host. *Am. J. Otolaryngol.* **2005***, 26*, 333-6.

426. Tilak, R.; Raina, P.; Gupta, S.K.; Tilak, V.; Prakash, P.; Gulati, A.K. Cutaneous zygomycosis: a possible postoperative complication in immunocompetent individuals. *Indian J. Dermatol. Venereol. Leprol.* **2009**,*75*,596-9

427. Tintelnot, K.; Nitsche, B. *Rhizopus oligosporus* as a cause of mucormycosis in man. *Mycoses* **1989***, 32*, 115-8.

428. Tiong, WH.; Ismael, T.; McCann, J. Post-traumatic and post-surgical *Absidia corymbifera* infection in a young, healthy man. *J. Plast. Reconstr. Aesthet. Surg.* **2006***, 59*, 1367-71.

429. Tomford, J.W.; Whittlesey, D.; Ellner, J.J.; Tomashefski, J.F Jr. Invasive primary cutaneous phycomycosis in diabetic leg ulcers. *Arch. Surg.* ***1980****, 115*, 770-1.

430. Tomita, H.; Muroi, E.; Takenaka, M.; Nishimoto, K.; Kakeya, H.; Ohno, H.; *et al*. *Rhizomucor variabilis* infection in human cutaneous mucormycosis. *Clin. Exp. Dermatol.* **2011***, 36*, 312-4.

431. Torell, J.; Cooper, B.H.; Helgeson, N.G. Disseminated *Saksenaea vasiformis* infection. *Am. J. Clin. Pathol.* **1981***, 76*, 116-21.

432. Trabelsi, H.; Neji, S.; Hadrich, I.; Sellami, M.; Khemakhem, N.; Sellami, H.; *et al*. Unusual case of otomycosis caused by *Saksenaea vasiformis*. *Med. Mycol. Case Rep.* **2020***, 27*, 68-71.

433. Trigg, M.E.; Comito, M.A.; Rumelhart, S.L. Cutaneous mucor infection treated with wide excision in two children who underwent marrow transplantation. *J. Pediatr. Surg.* **1996***, 31*, 976-7.

434. Trotter, D.J.; Gonis, G.; Cottrill, E.; Coombs, C. Disseminated *Saksenaea vasiformis* in an immunocompetent host. *Med. J. Aust.* **2008***, 189*, 519-20.

435. Tsoutsos, D.; Tsati, E.; Metaxotos, N.; Keramidas, E.; Rodopoulou, S.; Ioannovich, J. Extensive burn injury complicated by mucormycosis: a case report. *Ann. Burn Fire Disasters* **2001**, *14*, 126-8.

436. Tyll, T.; Lyskova, P.; Hubka, V.; Muller, M.; Zelenka, L.; Curdova, M.; *et al*. Early diagnosis of cutaneous mucormycosis due to *Lichtheimia corymbifera* after a traffic accident. *Mycopathologia* **2016***, 181*, 119-24.

437. Uçkay, I.; Chalandon, Y.; Sartoretti, P.; Rohner, P.; Berney, T.; Hadaya, K.; van Delden, C. Invasive zygomycosis in transplant recipients. Clin. Transplant. 2007, 21, 577-82.

438. Umbert, I.J.; Su, W.P. Cutaneous mucormycosis. *J. Am. Acad. Dermatol.* **1989***, 21*, 1232-4.

439. Vainrub, B.; Macareno, A.; Mandel, S.; Musher, D.M. Wound zygomycosis (mucormycosis) in otherwise healthy adults. *Am. J. Med.* **1988***, 84*, 546-8.

440. Vega, W.; Orellana, M.; Zaror, L.; Gené, J.; Guarro, J. *Saksenaea vasiformis* infections: case report and literature review. *Mycopathologia* **2006***, 162*, 289-94.

441. Veliath, A.J.; Rao, R.; Prabhu, M.R.; Aurora, A.L. Cutaneous phycomycosis (mucormycosis) with fatal pulmonary dissemination. *Arch. Dermatol.* **1976***, 112*, 509-12.

442. Venezio, F.R.; Sexton, D.J.; Forsythe, R.; Williams, M.; Reisberg, B. Mucormycosis after open fracture injury. *South Med. J.* **1985***, 78*, 1516-7.

443. Verma, R.; Nair, V.; Vasudevan, B.; Vijendran, P.; Behera, V.; Neema, S. Rare case of primary cutaneous mucormycosis of the hand caused by *Rhizopus microsporus* in an immunocompetent patient. *Int. J. Dermatol.* **2013***, 53*, 66-9.

444. Vernon, S.E.; Dave, S.P. Cutaneous zygomycosis associated with urate panniculitis. *Am. J. Dermatopathol.* **2006***, 28*, 327-30.

445. Vinay, K.; Chandrasegaran, A.; Kanwar, A.J.; Saikia, U.N.; Kaur, H.; Shivaprakash, M.R.; Dogra, S. Primary cutaneous mucormycosis presenting as a giant plaque: uncommon presentation of a rare mycosis. *Mycopathologia* **2014***, 178*, 97-101.

446. Viscoli, C.; Dodi, F.; Pellicci, E.; Ardizzone, G.; Soro, O.; Ceppa, P.; *et al*. *Staphylococcus aureus* bacteraemia, *Absidia corymbifera* infection and probable pulmonary aspergillosis in a recipient of orthotopic liver transplantation for end stage liver disease secondary to hepatitis C. *J. Infect.* **1997***, 34*, 281-3.

447. Visser, D.H.; van den Berg, Y.L.; van Furth, A.M.; Oomen, M.W.; Schouten-van Meeteren, A.Y.; Pajkrt, D.; *et al*. Diagnosis and treatment of cutaneous zygomycosis. *Pediatr. Infect. Dis. J.* **2007***, 26*, 1165-6.

448. Vitrat-Hincky, V.; Lebeau, B.; Bozonnet, E.; Falcon, D.; Pradel, P.; Faure, O.; *et al*. Severe filamentous fungal infections after widespread tissue damage due to traumatic injury: six cases and review of the literature. *Scand. J. Infect. Dis.* **2009***, 41*, 491-500.

449. Vulsteke, J.B.; Deeren, D. Cutaneous mucormycosis. *Transpl. Infect. Dis*. **2019**, 21, e13039.

450. Wall, S.J.; Lee, K.H.; Alvarez, J.D.; Bigelow, D.C. Quiz case 1. Cutaneous mucormycosis of the external ear. *Arch. Otolaryngol. Head Neck Surg.* **2000***, 126*, 236, 238-9.

451. Wang, Y.; Zhu, M.; Bao, Y.; Li, L.; Zhu, L.; Li, F.; *et al*. Cutaneous mucormycosis caused by *Rhizopus microsporus* in an immunocompetent patient: A case report and review of literature. *Medicine* **2018***, 97*, e11141.

452. Wang, X.; Ding, H.; Chen, Z.; Zeng, X.; Sun, J.; Chen, H.; Fu, M. CARD9 deficiency in a chinese man with cutaneous mucormycosis, recurrent deep dermatophytosis and a review of the literature. *Mycopathologia* **2020***, 185*, 1041-50.

453. Wang, J.; Harvey, C.M; Calhoun, J.H.; Yin, L.Y.; Mader, J.T. Systemic *Apophysomyces elegans* after trauma: case report and literature review. *Surg. Infect.* **2002***, 3*, 283-9.

454. Weddle, G.; Gandy, K.; Bratcher, D.; Pahud, B.; Jackson, M.A. *Apophysomyces trapeziformis* infection associated with a tornado-related injury. *Pediatr. Infect. Dis. J.* **2012***, 31,* 640-2.

455. Wee Liang, E.; Seow, Yen, T.; Ai Ling, T.; Yen Ee, T; Sze Hwa, T; Chun, AC; *et al*. Disseminated mucormycosis due to *Saksenaea vasiformis* complex in an immunocompetent adult with sustained response to posaconazole treatment. *Mycopathologia* **2020***, 185*, 577-81.

456. Wei, L.W.; Wang, H.; Song, Y.G.; Yu, J. Disfiguring *Mucor irregularis* infection cured by amphotericin B and itraconazole: a case report and treatment experience. *Mycopathologia* **2019***, 184*, 677-82.

457. Weinberg, WG.; Wade, BH.; Cierny, G 3rd.; Stacy, D.; Rinaldi, M.G. Invasive infection due to *Apophysomyces elegans* in immunocompetent hosts. *Clin. Infect. Dis.* **1993***, 17*, 881-4.

458. Weiss, S.C.; Moschella, S.L.; Kwan, T.; Craven, D.E. Cutaneous mucormycosis secondary to acquired reactive perforating collagenosis. *Cutis* **2003***, 72*, 119-23.

Articles included in the analysis for the systematic review

459. Weitzman, I.; Della-Latta, P.; Housey, G.; Rebatta, G. *Mucor ramosissimus* Samutsevitsch isolated from a thigh lesion. *J. Clin. Microbiol.* **1993***, 31*, 2523-5.

460. West, B.C.; Kwon-Chung, K.J.; King, J.W.; Grafton, W.D.; Rohr, M.S. Inguinal abscess caused by *Rhizopus rhizopodiformis*: successful treatment with surgery and amphotericin B. *J. Clin. Microbiol.* **1983***, 18*, 1384-7.

461. West, B.C.; Oberle, A.D.; Kwon-Chung, K.J. Mucormycosis caused by *Rhizopus microsporus* var. *microsporus*: cellulitis in the leg of a diabetic patient cured by amputation. *J. Clin. Microbiol.* **1995***, 33*, 3341-4.

462. Wickline, C.L.; Cornitius, T.G.; Butler, T. Cellulitis caused by *Rhizomucor pusillus* in a diabetic patient receiving continuous insulin infusion pump therapy. *South Med. J.* **1989***, 82*, 1432-4.

463. Wieden, M.A.; Steinbronn, K.K.; Padhye, A.A; Ajello, L.; Chandler, F.W. Zygomycosis caused by *Apophysomyces elegans*. J. Clin. Microbiol. 1985, 22, 522-6.

464. Wilcken, D.E.; Mackie, J.D.; Pussell, B.A.; Gatus, B.; Burke, D.J.; Palmer, F.J.; Warren, B.A. An unusual infection after renal transplantation. *Med. J. Aust.* **1986***, 145*, 513-7.

465. Wilson, W.; Ali-Osman, F.; Sucher, J.; Shirah, G.; Mangram A. Invasive fungal wound infection in an otherwise healthy trauma patient (Mucor Trauma). *Trauma Case Rep.* **2019***, 24*, 100251.

466. Wilson, C.B.; Siber, G.R.; O'Brien, T.F.; Morgan, A.P. Phycomycotic gangrenous cellulitis. A report of two cases and a review of the literature. *Arch. Surg.* **1976***, 111*, 532-8.

467. Wilson, M.; Robson, J.; Pyke, C.M.; McCormack, J.G. *Saksenaea vasiformis* breast abscess related to gardening injury. *Aust. N. Z. J. Med.* **1998***, 28*, 845-6.

468. Wilson, P.A. Zygomycosis due to *Saksenaea vasiformis* caused by a magpie peck. *Med. J. Aust.* **2008***, 189*, 521-2.

469. Wirth, F.; Perry, R.; Eskenazi, A.; Schwalbe, R.; Kao, G. Cutaneous mucormycosis with subsequent visceral dissemination in a child with neutropenia: a case report and review of the pediatric literature. *J. Am. Acad. Dermatol.* **1997***, 36*, 336-41.

470. Wollstein, R.; Palekar, A. Mucormycosis infection following intravenous access in the forearm. *Can. J. Plast. Surg.* **2010***, 18*, e30-2.

471. Woods, S.G.; Elewski, B.E. Zosteriform zygomycosis. *J. Am. Acad. Dermatol.* **1995***, 32*, 357-61.

472. Xia, Z.K.; Wang, W.L.; Yang, R.Y. Slowly progressive cutaneous, rhinofacial, and pulmonary mucormycosis caused by *Mucor irregularis* in an immunocompetent woman. *Clin. Infect. Dis.* **2013***, 56*, 993-5.

473. Xia, X.J.; Shen, H.; Liu, Z.H. Primary cutaneous mucormycosis caused by *Mucor irregularis*. *Clin. Exp. Dermatol.* **2015***, 40*, 875-8.

474. Xu, S.; Nambudiri, V.E.; Tahan, S.; Seo, S.J. Violaceous necrotic plaques on the leg of an immunosuppressed patient. Cutaneous mucormycosis. *J.A.M.A. Dermatol.* **2014***, 150*, 79-81.

475. Yacoub, A.; Soni, K.K.; Mojica, L.; Mai, J.; Morano, J.; Cruse, C.W.; *et al*. Primary gangrenous cutaneous mold infections in a patient with cancer and neutropenia. *Cancer Control.* **2016***, 23*, 265-71.

476. Yamaguchi, S.; Okubo, Y.; Katano, A.; Sano, A.; Uezato, H.; Takahashi, K. Primary cutaneous mucormycosis caused by *Mucor irregularis* in an elderly person. *J. Dermatol.* **2015***, 42*, 210-4.

Articles included in the analysis for the systematic review

477. Zachary, D.; Chapin, K.; Binns, L.; Tashima, K. Cutaneous mucormycosis complicating a polymicrobial wound infection following a dog bite. *Case Rep. Infect. Dis.* **2011***, 2011,* 348046.

478. Zaman, K.; Kaur, H.; Rudramurthy, S.M.; Singh, M.; Parashar, A.; Chakrabarti, A. Cutaneous mucormycosis of scalp and eyelids in a child with type I diabetes mellitus. *Indian J. Dermatol. Venereol. Leprol.* **2015***, 81*, 275-8.

479. Zhang, S.; Zhu, K.; Zhang, C. Successful treatment of a patient with cutaneous co-infection caused by *Mucor irregularis* and *Klebsiella pneumoniae*. *An. Bras. Dermatol.* **2020***, 95*, 623-6.

480. Zhao, L.; Wang, C.X.; Zhang, L.; Tu, X.A.; Wang, W.; Chen, Y.; Liu, L.S. Mucormycosis extending from the surgical wound to the transplanted kidney: case report and literature review. *Exp. Clin. Transplant.* **2012***, 10*, 403-5.

481. Zhao, Y.; Zhang, Q.; Li, L.; Zhu, J.; Kang, K.; Chen, L. Primary cutaneous mucormycosis caused by *Rhizomucor variabilis* in an immunocompetent patient. *Mycopathologia* **2009***, 168*, 243-7.

482. Zimhony, O.; Israeli, E.; Malnick, S.D.; Pansky, A.; Cohen, P.; Geltner, D. Zygomycotic gangrenous cellulitis in a patient with non-insulin dependent diabetes mellitus. *West J. Med.* **1998***, 168*, 192-4.

483. Zirak, C.; Brutus, J.P.; De Mey, A. Atypical cause of forearm skin ulceration in a leukaemic child: mucormycosis. A case report. *Acta Chir. Belg.* **2005***, 105*, 551-3.

484. Zizi, Yu.; Theodosakis, N.; Fitzpatrick, M.J.; Foreman, R.K.; Mackool, B. A 39-year-old male congenital tricuspid atresia patient who presented with a new axillary lesion after an orthotopic heart transplant. *Dermatopathology* **2019***, 6*, 220-4.

Articles included in the analysis for the systematic review

485. Zuglian, G.; Ripamonti, D.; Tebaldi, A.; Rizzi, M. Cutaneous mucormycosis by *Rhizopus arrhizus* treated with isavuconazole as first line therapy: a case report. *Med. Mycol. Case Rep.* **2019***, 26*, 42-3.
